# Species Identification in Malaise Trap Samples by DNA Barcoding Based on NGS Technologies and a Scoring Matrix

**DOI:** 10.1371/journal.pone.0155497

**Published:** 2016-05-18

**Authors:** Jérôme Morinière, Bruno Cancian de Araujo, Athena Wai Lam, Axel Hausmann, Michael Balke, Stefan Schmidt, Lars Hendrich, Dieter Doczkal, Berthold Fartmann, Samuel Arvidsson, Gerhard Haszprunar

**Affiliations:** 1 SNSB, Bavarian State Collection of Zoology, Münchhausenstrasse 21, 81247, München, Germany; 2 LGC Genomics GmbH, Ostendstraße 25, TGS Haus 8, 12459, Berlin, Germany; 3 GeoBio-Center der LMU München, München, Germany; Consiglio Nazionale delle Ricerche (CNR), ITALY

## Abstract

The German Barcoding initiatives BFB and GBOL have generated a reference library of more than 16,000 metazoan species, which is now ready for applications concerning next generation molecular biodiversity assessments. To streamline the barcoding process, we have developed a meta-barcoding pipeline: We pre-sorted a single malaise trap sample (obtained during one week in August 2014, southern Germany) into 12 arthropod orders and extracted DNA from pooled individuals of each order separately, in order to facilitate DNA extraction and avoid time consuming single specimen selection. Aliquots of each ordinal-level DNA extract were combined to roughly simulate a DNA extract from a non-sorted malaise sample. Each DNA extract was amplified using four primer sets targeting the CO1*-5’* fragment. The resulting PCR products (150-400bp) were sequenced separately on an Illumina Mi-SEQ platform, resulting in 1.5 million sequences and 5,500 clusters (coverage ≥10; CD-HIT-EST, 98%). Using a total of 120,000 DNA barcodes of identified, Central European Hymenoptera, Coleoptera, Diptera, and Lepidoptera downloaded from BOLD we established a reference sequence database for a local *CUSTOM* BLAST. This allowed us to identify 529 Barcode Index Numbers (BINs) from our sequence clusters derived from pooled Malaise trap samples. We introduce a scoring matrix based on the sequence match percentages of each amplicon in order to gain plausibility for each detected BIN, leading to 390 high score BINs in the sorted samples; whereas 268 of these high score BINs (69%) could be identified in the combined sample. The results indicate that a time consuming presorting process will yield approximately 30% more high score BINs compared to the non-sorted sample in our case. These promising results indicate that a fast, efficient and reliable analysis of next generation data from malaise trap samples can be achieved using this pipeline.

## Introduction

Faunal monitoring is the most viable way to attest the general ecosystem health [1]. It enables scientists to detect invasive species, to study spatial and temporal dynamics of species within an ecosystem, and to define areas for conservation priority settings, among other important biodiversity and ecosystem management decisions. Insects are a major component of biodiversity in virtually all terrestrial ecosystem [2], making them very important for environmental impact assessment [3]. In this context, malaise traps are an often used, standardized method for sampling flying insects [4–7], being also used to inventory biodiversity worldwide (www.globalmalaise.org). However, for some hyperdiverse insect orders, in particular Hymenoptera and Diptera, even experts will need several weeks to identify samples just to genus level. Moreover, it is often impossible to collaborate with dozens of taxonomists for multiple taxa [8]. Species level keys are rare and/or difficult to use; moreover, they usually only deal with selected groups of geographically restricted fauna making it difficult for non-specialists to identify the specimens. One common way to bypass these problems is sending the specimens to taxonomic specialists of particular groups, but this process is time consuming and depends on the availability of the person who will receive the material. It is also important to consider the damage or loss risks of postage. Another impeditive factor is the lack of taxonomic expertise for certain groups of insects, usually addressed as the “taxonomic impediment” [9–10]. To circumvent these problems, supraspecific taxa or morphospecies sorting are often used in large surveys, often resulting in highly inaccurate estimations of species diversity, however. More importantly, such data are high ambiguous and thus neither scientific nor sustainable [11–12].

High throughput DNA sequencing might here offer an alternative to generate more objective (i.e. checkable), globally accessible data. Some recent studies discussed the drawbacks when using various gene markers (e.g. 18S, 16S, *cytB*) for biodiversity assessment of benthic and marine ecosystems, whereas detection of OTUs was possible, a delineation of species was not perfectly applicable due to lack of available marker libraries [13–15]. Especially for ecosystems like soil or benthos the reference libraries are still not representative, whereas first pioneering studies analyzing selected taxa have been published [16–22]. Furthermore, the necessity of generation and maintenance of individually barcoded and curated specimens in museum collections to link metabarcoding sequences to species was discussed [23]. Within this context the detection and identification of invasive alien species, which have their origin in another less well curated origin, are also presented as disadvatages of metabarcoding studies [24]. The use of next generation sequencing (NGS) within the DNA barcoding framework provides a promising tool to analyze extremely large amounts of specimens economically [25–31].

More than a decade after the onset of DNA barcoding [32], approximately 380,000 barcode clusters have been uploaded to the Barcoding of Life Database (BOLD– www.boldsystems.org) [33]. This reference library is particularly comprehensive for Central Europe, and in particularly for Germany. Since 2009, two major barcoding initiatives were coordinated or supported by scientists of the Bavarian State Collection of Zoology (ZSM, Munich, Germany): the Barcoding Fauna Bavarica project (BFB– www.faunabavarica.de) and the German Barcode of Life Project (GBOL– www.bolgermany.de). The German barcoding projects aim to assemble a comprehensive DNA barcode library for all German animal species in the framework of the International Barcode of Life (iBOL) campaign. For that purpose, scientists of the ZSM are working in close cooperation with the Biodiversity Institute of Ontario (BIO, Guelph, Canada). Until now, the German Barcode initiatives (BFB & GBOL) have generated a reference library for more than 16,000 animal species, with focal groups being Coleoptera [34], bees [35], Neuroptera [36], Heteroptera [37], and Lepidoptera [38–39]. DNA barcoding relies on the existence of such comprehensive reference libraries of species identified and species hypotheses updated by expert taxonomists. In BOLD, similar CO1 barcode sequences are assigned a globally unique identifier (Barcode Index Number, BIN, [33, 40]). This system offers a suitable species-group proxy, if taxonomic information is still lacking (e.g. for many Diptera).

Here, we aim to evaluate the comprehensiveness of the DNA Barcode reference library, especially for Coleoptera, Diptera, Hymenoptera, and Lepidoptera using 120,000 DNA Barcode sequences of species with a corresponding BIN in the BOLD database. We also tested the plausibility of high confidence candidate BINs for species identification by applying four primers targeting the CO1 fragment. Finally we also tested the importance of a pre-sorting process to yield better species assessment.

## Materials and Methods

### Ethics and legal statements

Field work permits were issued by the responsible state environmental office of Bavaria [Bayerisches Staatsministerium für Umwelt und Verbraucherschutz, Munich, Germany, project: “Barcoding Fauna Bavarica”, reference number 62e-U8645.8-2008/3-17]. The study sites comprise state forests, public land and protected areas. We confirm that the field studies for the present contribution did not involve any protected species by European or national laws.

### Collection locality

The malaise trap was set near Oberammergau in the Bavarian Alps and operated from 6^th^ until 18^th^ August 2014. It was situated at 1,010 meters elevation in an area covered by anthropogenic nutrient poor grass vegetation (*Nardetum*) close to the edge of a mixed forest (47.61707°N 11.05900°E).

### Taxon sampling & sorting

**Fig 1:**
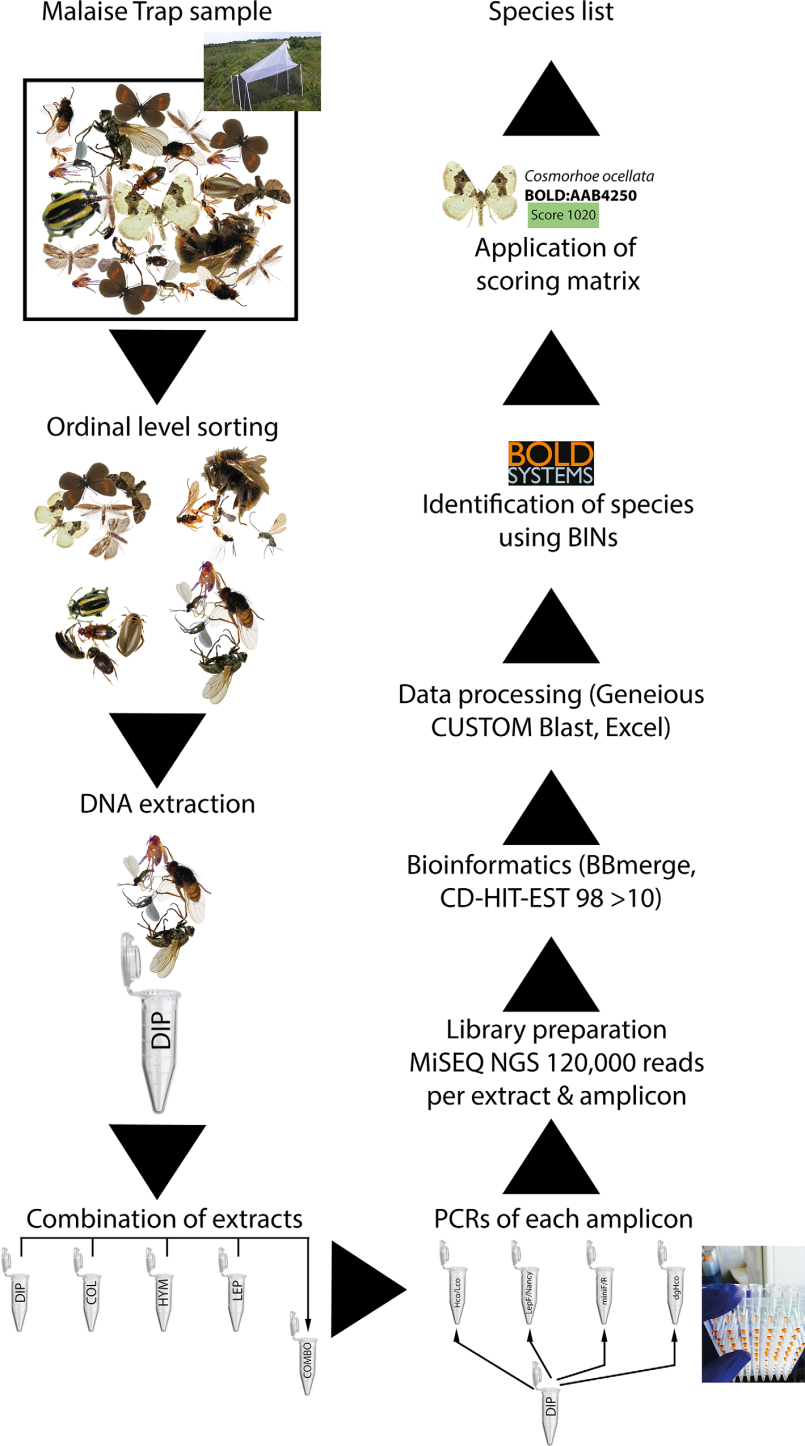
Visualization of the study workflow.

Samples were stored in 80% EtOH in a freezer until the insects from this trap were sorted to ordinal level using a Leica MZ9.5 stereo microscope. After sorting, specimens were transferred into 96% EtOH. The sorting of the ca. 5,000 specimens took about 60 hours, and contained predominantly Coleoptera (ca. 500 specimens), Hymenoptera (ca. 1,500 specimens), and Diptera (ca. 2,000 specimens). These highly represented orders were kept separated while the orders represented by few specimens were combined in groups (**Table 1, Fig 1**).

**Table 1:** Arthropod orders sorted and combined by sample number.

Sample Number	Arthropod order
**1**	Aranaea & Opiliones
**2**	Collembola
**3**	Dermaptera & Blattodea
**4**	Mecoptera & Neuroptera
**5**	Pscoptera
**6**	Trichoptera
**7**	Hemiptera
**8**	Coleoptera
**9**	Orthoptera
**10**	Lepidoptera
**11**	Hymenoptera
**12**	Diptera
**13**	Combined fraction of numbers 1–12

### DNA extraction

DNA extraction was performed using the DNEasy tissue kit (Qiagen, Hilden–Germany). A mixture of Proteinase K and lysis buffer was pre-mixed in different volumes to account for the difference in the volume of the sorted specimens. To facilitate the contact between lysis buffer and tissue, we briefly macerated the arthropods using sterilized forceps. Tissue lysis was performed at 56°C for eight hours, and the samples were mixed by inverting about 10 times every hour to improve the lysis buffer reaction. A total volume of between 150μL to 600μL of lysate (depending on the number of specimens and their size in each sorted group) was used for DNA extraction following the manufacturer’s specifications. In order to simulate an unsorted sample, we mixed an aliquot of 20μL from each separately extracted group. Extracted DNA was then sent to LGC Genomics (Berlin–Germany) for amplification and NGS analyses.

### Amplification of CO1

We used 5ng of DNA extract for amplification of the barcoding region of the cytochrome c oxidase subunit I gene (CO1). For PCR amplification, we used the MyTaq DNA Polymerase kit (Bioline, Luckenwalde–Germany). For each reaction, 1.5U of MyTaq were pre-mixed with 20μL MyTaq buffer containing 15pmol of the forward and reverse primer, 2μL BioStabII PCR Enhancer (Sigma Aldrich, St. Louis–United States) and 1μL DMSO. We used four different amplicons targeting the CO1 gene (**Table 2**). For PCR of each DNA extract sample, a unique 8 base-barcode tag was used in the forward and reverse primer. DNA concentrations of amplified amplicons were checked via agarose gel electrophoresis. Approximately 20ng of amplified PCR product of each sample were transferred into amplicon-pools of up to 48 parallel samples. Samples yielding a lower amplicon concentration were amplified for another 5 cycles in an additional PCR reaction.

**Table 2:** Primers and corresponding PCR conditions used in this study.

Amplicon	Sequence	Reference	PCR conditions
**Hco2198**	5’—TAA ACT TCA GGG TGA CCA AAA ATC A—3’	[47]	2’::94°C– 5x[30”:94°C– 40”:45°C– 1’:72°C]– 35x[30”:94°C– 40”:50°C– 1’:72°C]– 10’:72°C
**Lco1490**	5’—GGT CAA CAA ATC ATA AAG ATA TTG G—3’	[47]	
**LepF**	5’—ATT CAA CCA ATC ATA AAG ATA TTG G—3’	[48]	2’:94°C– 5x[1’:94°C– 90”:45°C– 90”:72°C]– 35x[1’:93°C– 90”:50°C– 90”:72°C]– 10’:72°C
**Nancy**	5’—CCT GGT AAA ATT AAA ATA TAA ACT TC—3’	[49]	
**miniF**	5’—GAA AAT CAT AAT GAA GGC ATG AGC—3’	[50]	2’:95°C– 5x[1’:95°C– 1’:46°C– 30”:72°C]– 35x[1’:95°C –1’:53°C– 30”:72°C]– 5’:72°C
**miniR**	5’—TCC ACT AAT CAC AAR GAT ATT GGT AC—3’	[50]	
**dgHco**	5’—TAA ACT TCA GGG TGA CCA AAR AAY CA—3’	[14]	2‘:96°C– 3x[15‘‘:96°C– 30‘‘:48°C– 90‘‘:65°C]– 30x[15‘‘:96°C– 30‘‘:55°C—90‘‘:65°C]– 10’:72°C
**mlCOIintF**	5’—GGW ACW GGW TGA ACW GTW TAY CCY CC—3’	[14]	

### Next Generation Sequencing

Amplicon-pools were cleaned using a 1.2 fold volume of Ampurebeads (Beckman Coulter, Pasadena, United States) to eliminate mispriming products, primer dimers and residual primers. To remove protein residuals and to concentrate the amplicon-pool, samples were cleaned using a MinElute column (Qiagen, Hilden, Germany). We used 100ng of the cleaned amplicon-pools for construction of the Illumina libraries using the Ovation Rapid DR Multplex System 1–96 kit (NuGEN, San Carlos, United States). Illumina libraries were size-selected via preparative gel electrophoresis and subsequently sequenced on an Illumina MiSeq using V3 chemicals.

### Pre-processing of sequence data

Sample data was obtained by sorting the paired sequence reads of each amplicon pool on the individual sample inline barcode (present before the amplicon primer sequence). Subsequently, the reads were screened for remnant sequencing adapter sequences and clipped accordingly. Finally, all reads were filtered on the presence of valid primer sequence combinations and all sequences are turned into the Fwd-Rev primer direction (as the direction of sequencing is random). For the amplicons shorter than 570 base pairs (bp) the paired reads were combined into single fragments with BBmerge v.34.48 (http://sourceforge.net/projects/bbmap/); for the longer amplicons the forward and reverse reads were joined with extra Ns added between the sequences to make up for the expected total length. All pre-processing steps described here were carried out with proprietary software from LGC Genomics (LGC Genomics, Berlin, Germany), unless where specific software packages are mentioned.

### Sequence clustering

All nucleotide sequences obtained from all samples were clustered per amplicon with CD-HIT-EST v4.6.1-2012-08-27 (http://www.ncbi.nlm.nih.gov/pubmed/23060610) on 98% sequence identity. The most abundant sequences from each cluster were selected as representative sequences, and were used in all subsequent analyses.

### Data processing and scoring

In order to create a database that combines the sequences with the respective BIN, we downloaded public and privileged sequences and specimen data of approximately 120,000 reliably identified species of Coleoptera, Diptera, Hymenoptera and Lepidoptera from the BOLD projects of the BFB and GBOL projects. Sequences and corresponding BINs were merged in Microsoft Excel using the BOLD processID as unique identifier.

We created an input FASTA file to use it as a *CUSTOM BLAST* (options: Megablast, Results as Hit table, Maximum Hits allowed 1) database in Geneious v8.0.3 (Biomatters, Auckland—New Zealand). Consensus cluster sequences from each insect order amplified with the four amplicons received from LGC Genomics were used as input files for the *CUSTOM BLAST* search in Geneious. Results of the BLAST search were exported as a csv file and further processed in Excel. We copied the BINs, the sequence identities and the query numbers from the result table. This data was included to the results of sequence clusters detected in each group sample (including the combined sample) for each amplicon. In order to account for sequencing errors and within-species variations, we solely included BLAST results with a minimum of 97% sequence identity for further analyses. We created four categories based on the sequence identity percentage to score the sequence identity confidence (**Table 3**).

**Table 3:** Categories of scoring according to the sequence identity percentage.

Interval	Score
97.00–97.99%	70
98.00–98.99%	150
99.00–99.99%	240
100%	340

**Fig 2:**
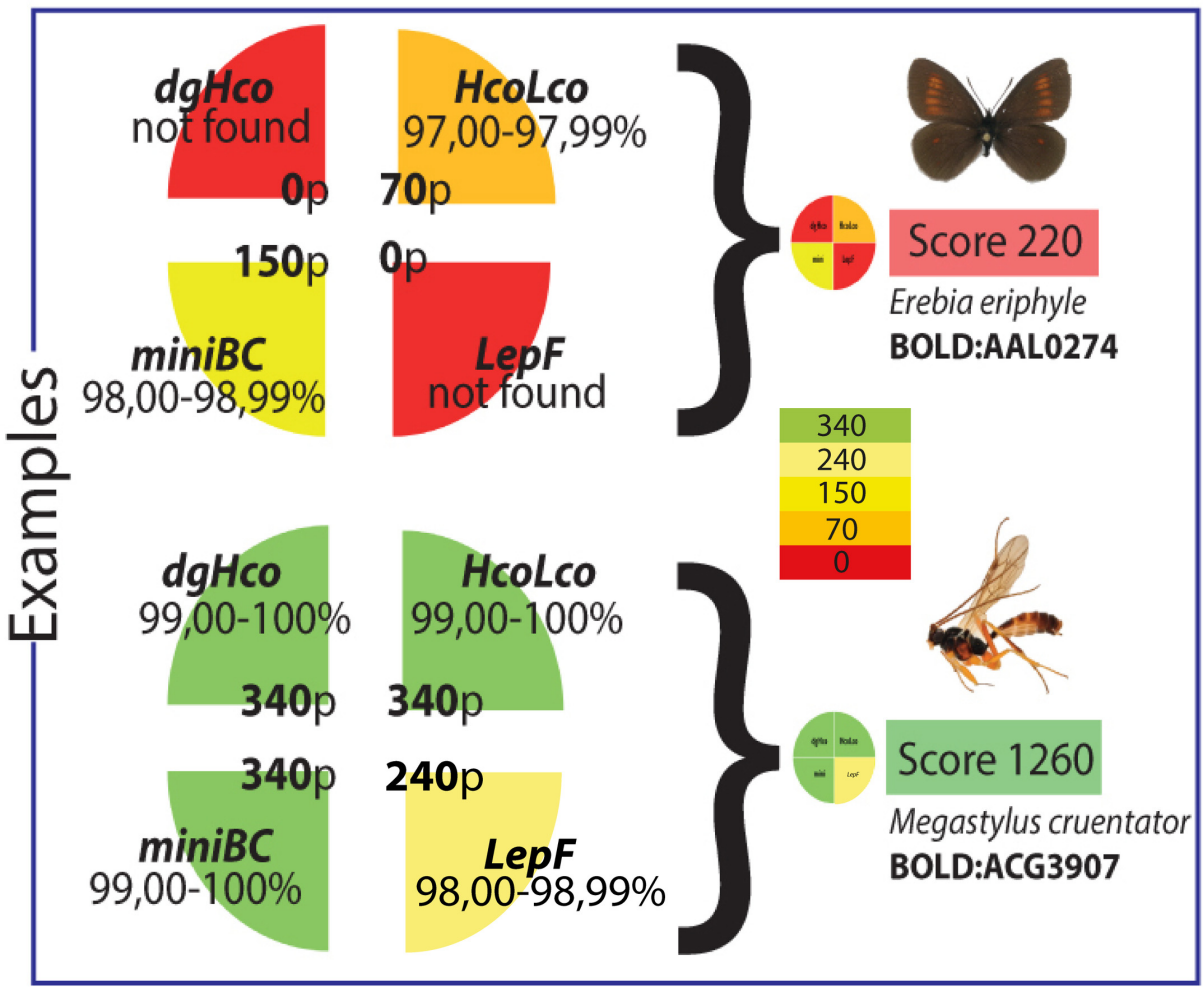
Examples of the scoring scheme used in this study. The upper Lepidopteran species (*Erebia eriphyle*, Fryer 1839) was not included into further analyses, as its score summed up to only 220. The lower Hymenopteran species (*Megastylus cruentator*, Schiødte 1839) represents a “high score BIN” with a total of 1260 points. Gradient code illustrates the used color for percentage values detected for each amplicon.

The results of each primer were scored and summed up to create an overall confidence index for each BIN. The highest possible score is 1360 resulting of 100% sequence identity match for all four primers used, whereas the lowest score is 70, in case a sequence with less than 98% identity is detected only by one amplicon. To avoid including BINs with the lowest confidence score for all amplicons (97%) yielding in 280 points, we eliminated all results with a score lower than 300 points (**Fig 2**).

## Results

**Fig 3:**
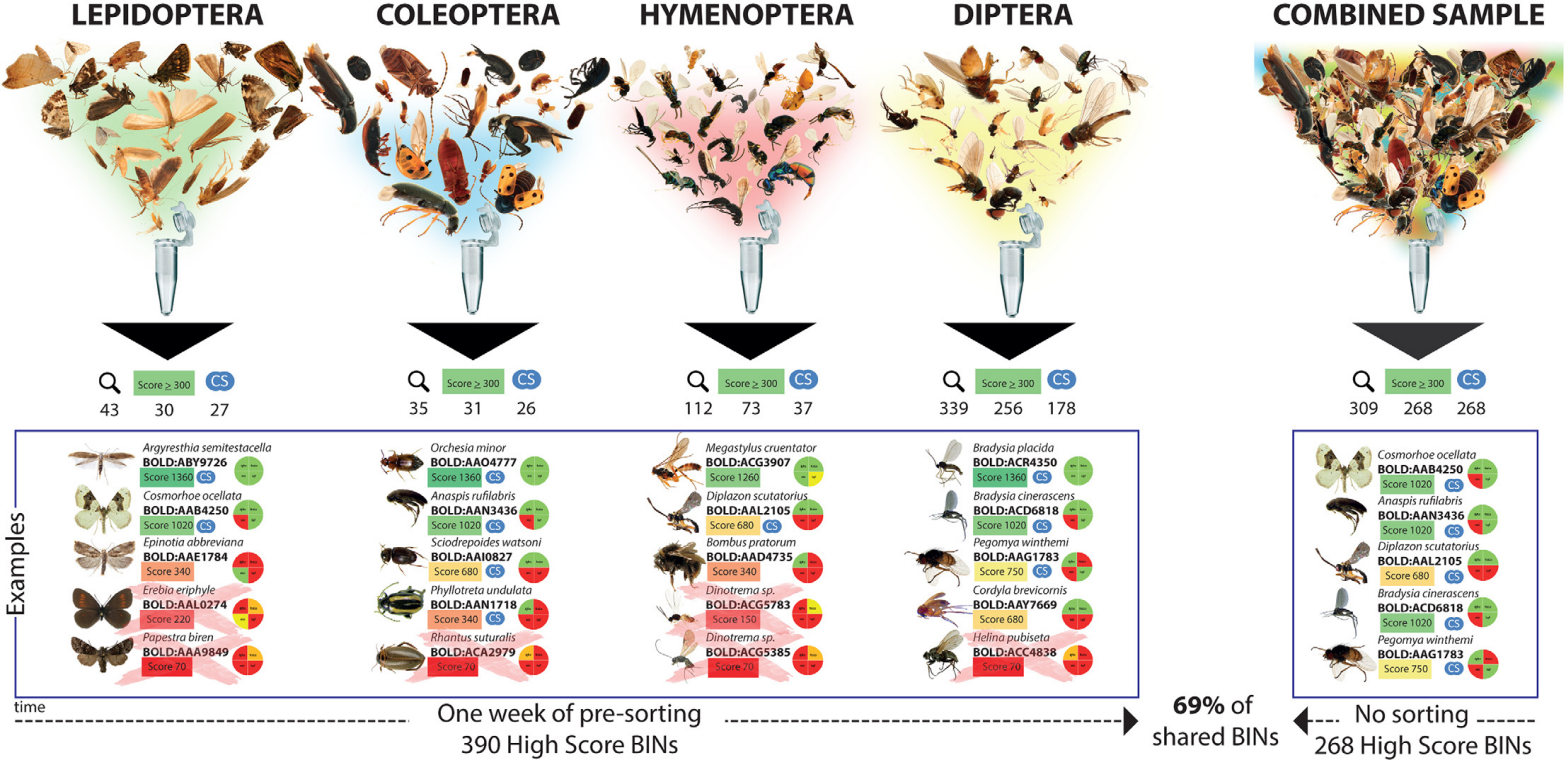
Results of the four ordinal level sorted arthropod orders and the combined fraction. Magnifying glasses represent total number of detected BINs, the Score ≥ 300 symbol represents the total number of detected BINs with a score ≥ 300 within the sample, the CS symbol represents number of shared BINs within the single ordinal level sorted and the combined fraction.

**Fig 4:**
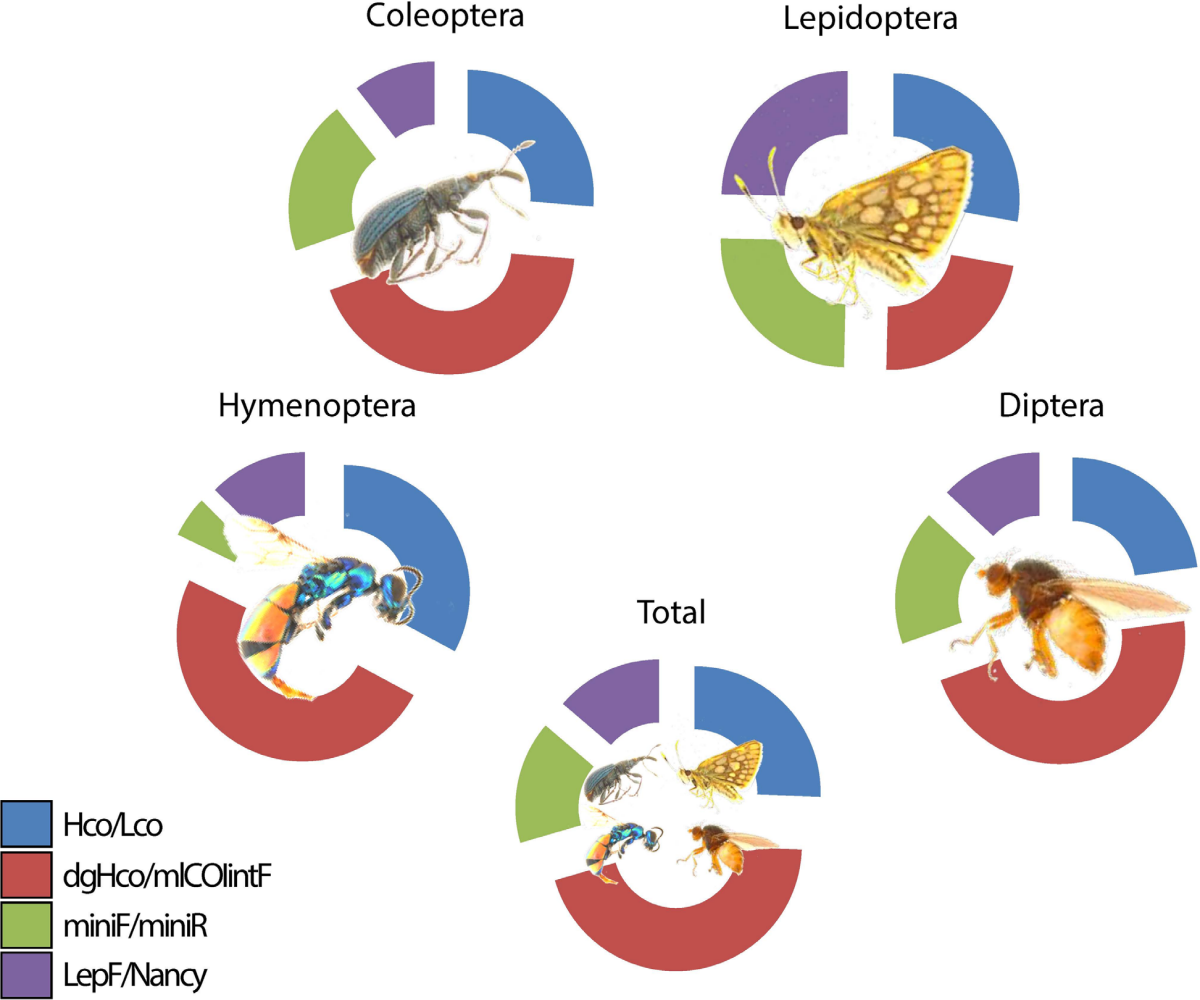
Primer efficiency for CO1 amplification of the four ordinal level sorted arthropod orders.

The amplified PCR products (150–400 bp) resulted in 1.5 million sequences, out of which 5,500 sequence clusters (coverage ≥10; CD-HIT-EST, 98%) were obtained and blasted using the *CUSTOM BLAST* against 120,000 DNA barcodes of reliably identified specimen, resulting in the detection of a total of 529 BINs fitting the selection criterion of at least 97% sequence identity (**S1 Table, S2 Table, S1 Fig, S1 File**). For Coleoptera we detected a total of 35 BINs, 31 (89%) of which were scored with more than 300 points. We detected a total of 339 BINs within the Dipteran sample, 256 (75%) of which were scored with more than 300 points. A total of 43 Lepidopteran BINs was detected, 30 (70%) of them were scored with more than 300 points. For Hymenoptera we were able to detect a total of 112 BINs, 73 (65%) of which reached a score higher than 300 points. To summarize the results of the different amplicons, 390 of the BINs were identified as “high score BINs” with a score ≥ 300 points (another 139 BINs with a lower score were excluded) (**Fig 3**). The dgHCO primer was most efficient for all orders, especially for Coleoptera, Diptera and Hymenoptera. The second most efficient primer was HCO for all orders studied. For Lepidoptera all four primers had similar efficiency (**Table 4**, **Fig 4**). The primer efficiency was the same in the combined sample.

**Table 4:** Efficiency of amplicons used.

	Hco	dgHco	miniBC	LepF
Lepidoptera	28%	23%	25%	25%
Hymenoptera	33%	49%	5%	13%
Coleoptera	26%	43%	20%	11%
Diptera	23%	47%	17%	13%
total	26%	45%	16%	14%

Number of BINs (compared to the total) is displayed as percentage values.

In order to discern BINs that could be identified without a time consuming pre-sorting process, all BINs were detected and checked for sufficient score values and then compared with the combined sample. A total of 309 BINs (59% of the previously detected 529 BINs in the sorted samples) were detected in the combined sample, whereas 268 of these BINs had a score of at least 300 points, corresponding to 69% of the 390 high score BINs detected within the various presorted fractions.

## Discussion

Central to all comprehensive biodiversity assessments, is the ability to monitor species compositions of bulk samples in an efficient, accurate and cost effective way [41]. The development of such tool is vastly needed and is becoming more and more important as the detection of certain bio-indicators or invasive species is crucial [13–15, 30, 42–44]. Although such a tool for fast and high throughput analysis is urgently required for various fields of applications (such as environmental DNA surveys, soil compositions, faeces, detection of food compositions, river benthos analysis, as well as zooplankton, phytolankton and benthos from the marine realm), compiling a complete list of all species represented in a bulk sample remains a challenging task for several reasons. Malaise traps are a common tool for monitoring ecosystem compositions such as arthropod biodiversity, but the sheer amount of specimens collected in addition to lack of taxonomists to identify the material in an applicable level makes it very time consuming. That is why some studies are forced to use parataxonomic approaches to overcome those difficulties. Facing this situation NGS appears as an alternative to speed up the conventional process of ecological investigations, individual specimen isolation and identification, thus allowing a more precise biodiversity estimation of multiple taxa in a bulk mixture [31].

In regions such as Central Europe, where comprehensive DNA libraries for many taxa are available and identification of species using BINs is applicable for the local fauna (see for recent local fauna Barcoding studies: [35–39, 45–46]) the procedure of species delineation in a bulk sample using NGS is a suitable instrument.

Using four primers targeting the CO1 barcode region and applying the scoring matrix introduced in this study, we were able to check multiple results for the detected BINs resulting in a high level of certainty. Although NGS comprises a fast and efficient tool for monitoring biodiversity within a bulk sample, the use of only one primer could lead to insufficient results. All BINs that were detected with less than 100% sequence identity match for one primer needed to be recovered with at least two primers (e.g. having 99% for one primer and 97% for a second one) in order to reach the minimum score of 300 points, enhancing the robustness of each detected BIN. By checking the BINs with score values lower than 300 points, we mostly discovered species within these low score BINs, which are not typical for this habitat type (e.g. warm loving species, alpine species; e.g. *Pelosia obtusa*, Herrich-Schäffer, 1847) or non-local species (from Austria, Switzerland or France; e.g. *Euchloe sp*.). These findings underline the benefits of the use of multiple primers and the scoring system.

In this study we tried to quantify necessity of presorting samples to accurate BIN identification, we compared the results from a presorted sample to that of an unsorted sample. By incorporating the presorting step, we were able to recover 390 high score BINs, representing 31% more than the 268 high score BINs recovered in the combined sample (**Fig 2**). Despite the advantage of increased capture efficiency, the presorting procedure requires time and at least parataxonomic expertise to be done properly. Therefore, the decision of presorting for a mass sample should be made depending on the expected diversity of taxa, the availability of time, personnel and funding. As discussed before, the fact that the combined sample resulted in fewer high score BINs than the sorted sample, could be attributed to the number of reads and the amount of DNA in the combined sample in comparison to the presorted groups.

Another artifact that could have caused the difference in number of high score BINs identified is the uneven sequencing of the different types of samples. An equal number of 120,000 reads per amplicon (rpa) was performed for each group (Coleoptera, Diptera, Lepidoptera, Hymenoptera and the combined sample), resulting in a total of 480,000 rpa for the sorted samples, whereas only a total of 120,000 rpa for the combined sample were produced. As the combined sample was comprised of eight additional arthropod groups, the massive amount of target DNA could cause effects of primer competition resulting in a smaller read capacity proportional to DNA diversity. That could explain the differences between the amount of BINs found with and without sorting. Clearly, the advantage and effectiveness of the presorting procedure needs further and more explicit testing.

One factor that should also be considered is the various relatives amount of DNA extracted from each individual within a trap sample, which differ enormously in body size, for example large bumblebees (*Bombus*) versus tiny fairy flies (Mymaridae). The different amount of DNA available to be amplified in every single PCR reaction is very likely to influence the outcome of the NGS experiments [15]. Therefore, although we had visual confirmation of a big diversity of small specimens (e.g. Microhymenoptera), this diversity was not detected in either of the samples (presorted or combined). If a sorting by body size is responsible for this effect or if this might have other related issues as primer specification needs to be addressed in a future experiment. No size separation was performed here, as the goal of this study was to compare the effects of presorting versus no sorting and to test primer efficiency of the four amplicons used.

## Conclusions & Outlook

As also shown in other recent studies, NGS techniques provide a fast and cost efficient way to sequence thousands of specimens at once [25–31]. The novel scoring matrix introduced here provides a fast, efficient and reliable method to process malaise trap samples using NGS sequencing, as it increases the plausibility for each identified CO1 species cluster using four amplicons. The results indicate that if time, money, and personnel are limited, the presorting procedure can be excluded, if this is economically necessary. Some technical issues should be observed for future study designs. The amount of required reads for sufficient sampling should be planned adequately and proportionally taking into account different types and concentrations of DNA within different samples. If the specimens are sufficiently different in size, a procedure of sorting by size is recommended, to normalize the amount DNA contributed by each specimen in the extracts.

The diversity we here recovered using the NGS approach mostly agrees with the expected diversity estimates, conducted by the coauthors and their high experience of this kind of environment. The results of the NGS experiments underline the comprehensiveness for of the DNA Barcoding library for most groups studied here. We only invested approximately 14 man working days for the whole process of pre-sorting, laboratory work and data analysis, a small amount of work compared to the time necessary to carry out traditional alpha taxonomical methodologies. Furthermore, having a robust and comprehensive reference library at hand facilitates a precise delineation of species diversity in relation to the parataxonomic approach. All in all, we have demonstrated that comprehensive biodiversity assessments can be achieved accurately, efficiently and cost effectively through the use of NGS and thoughtful experimental design. However, additional future investigations with a more extensive study design, more malaise traps and a higher level of presorting efforts would be beneficial to improve the methods reported in this study.

## Supporting Information

S1 Fig Percentages of insect families discovered in the malaise trap sample. (TIF)

S1 File NGS data and Results. (ZIP)

S1 Table List of Barcode Index Numbers (BINs) detected for each insect order. The score of each BIN as well as the detection within the sorted and/or the combined sample are indicated.
(DOCX)

S2 Table List of all families per insect order studied and number of family representatives detected for each order. (DOCX)
