# Supplementary figures and images for: Species Identification in Malaise Trap Samples by DNA Barcoding Based on NGS Technologies and a Scoring Matrix
Source: PLoS One. 2016 May 18;11(5):e0155497. doi: 10.1371/journal.pone.0155497 (PMC4871420; doi:10.1371/journal.pone.0155497)

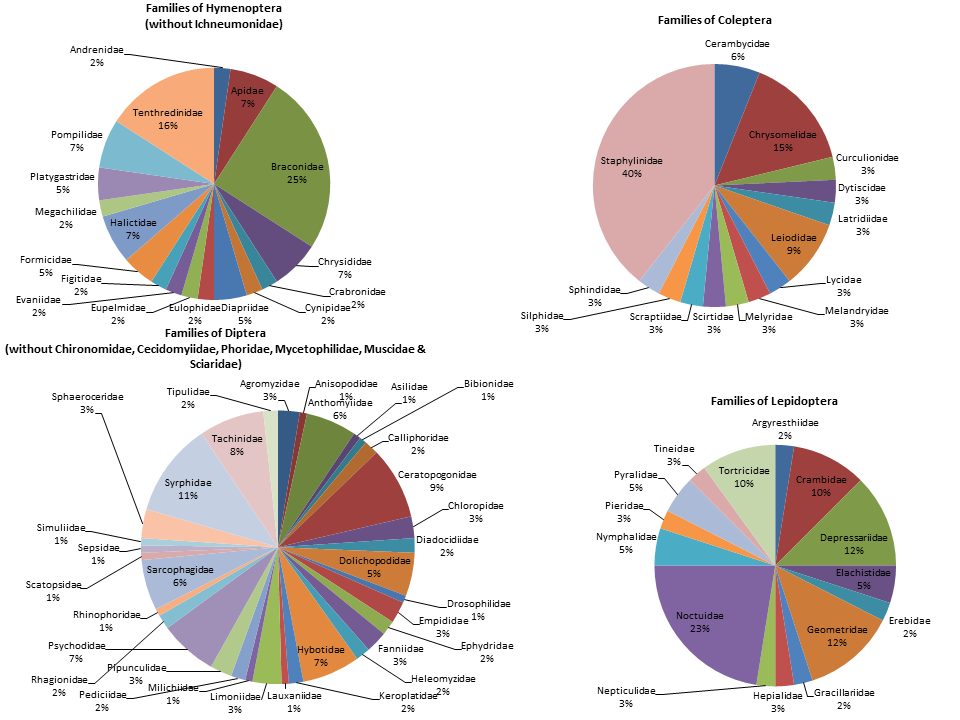

Supplement: S1 Fig — (TIF) [file pone.0155497.s001.tif]
