# Supplementary material for: Species Identification in Malaise Trap Samples by DNA Barcoding Based on NGS Technologies and a Scoring Matrix
Source: PLoS One. 2016 May 18;11(5):e0155497. doi: 10.1371/journal.pone.0155497 (PMC4871420; doi:10.1371/journal.pone.0155497)
Supplement: S1 Table — The score of each BIN as well as the detection within the sorted and/or the combined sample are indicated. (DOCX) [file pone.0155497.s003.docx]

**S1 Table . List of Barcode Index Numbers (BINs) detected for each insect order.** The score of each BIN as well as the detection within the sorted and/or the combined sample are indicated.

| Familiy | Species identification – |  | BIN | Score | Sorted | Combined |
| --- | --- | --- | --- | --- | --- | --- |
|  | **Lepidoptera** |  |  |  |  |  |
| Argyresthiidae | *Argyresthia semitestacella* | Curtis, 1833 | BOLD:ABY9726 | 1360 | X | X |
| Tortricidae | *Dichelia histrionana* | Frölich, 1828 | BOLD:ACF5563 | 1260 | X | X |
| Geometridae | *Scotopteryx chenopodiata* | Linnaeus, 1758 | BOLD:AAB5023 | 1260 | X | X |
| Noctuidae | *Mesapamea secalella* | Remm 1983 | BOLD:AAB2749 | 1260 | X | X |
| Tortricidae | *Pandemis heparana* | Schiffermüller, 1776 | BOLD:AAA9254 | 1260 | X | X |
| Nymphalidae | *Erebia aethiops* | Esper, 1777 | BOLD:AAB3455 | 1160 | X | X |
| Crambidae | *Eudonia truncicolella* | Stainton, 1849 | BOLD:AAB1558 | 1070 | X | X |
| Elachistidae | *Elachista canapennella* | (Hübner, 1813) | BOLD:AAB7810 | 1020 | X | X |
| Noctuidae | *Autographa gamma* | Linnaeus, 1758 | BOLD:AAB4345 | 1020 | X | X |
| Geometridae | *Cosmorhoe ocellata* | Linnaeus, 1758 | BOLD:AAB4250 | 1020 | X | X |
| Depressariidae | *Depressaria douglasella* | Stainton, 1849 | BOLD:ABZ6022 | 920 | X |  |
| Crambidae | *Eudonia lacustrata* | Panzer, 1804 | BOLD:AAB3829 | 830 | X | X |
| Hepialidae | *Triodia sylvina* | (Linnaeus, 1761) | BOLD:AAD0500 | 820 | X | X |
| Geometridae | *Peribatodes secundaria* | Denis & Schiffermüller, 1775 | BOLD:AAC2923 | 820 | X | X |
| Crambidae | *Pyrausta purpuralis* | Linnaeus, 1758 | BOLD:AAB6531 | 820 | X | X |
| Geometridae | *Colostygia olivata* | Schiffermüller, 1775 | BOLD:AAB9509 | 720 | X | X |
| Gracillariidae | *Phyllonorycter maestingella* | (Müller, 1764) | BOLD:AAL6962 | 340 | X |  |
| Tortricidae | *Epinotia abbreviana* | Fabricius, 1794 | BOLD:AAE1784 | 340 | X | X |
| Nepticulidae | *Stigmella regiella* | Herrich-Schäffer, 185? | BOLD:ACG9440 | 240 | X | X |
| Noctuidae | *Graphiphora augur* | Fabricius, 1781 | BOLD:ACF0935 | 150 | X |  |
| Pyralidae | *Assara terebrella* | Zincken, 1818 | BOLD:ACA2238 | 150 | X | X |
| Pieridae | *Euchloe* sp | Hübner, 1819 | BOLD:ABZ3927 | 150 | X | X |
| Nymphalidae | *Argynnis niobe* | (Linnaeus, 1758) | BOLD:ABY9182 | 150 | X |  |
| Depressariidae | *Depressaria beckmanni* | Heinemann, 1870 | BOLD:ABA1413 | 150 | X |  |
| Geometridae | *Scopula bigeminata* | Warren, 1897 | BOLD:AAU3280 | 150 | X |  |
| Erebidae | *Pelosia obtusa* | Herrich-Schäffer, 1847 | BOLD:AAQ2315 | 150 | X |  |
| Pyralidae | *Pima boisduvaliella* | Guenée, 1845 | BOLD:AAQ0520 | 150 | X |  |
| Noctuidae | *Conistra ragusae* | Failla-Tedaldi, 1890 | BOLD:AAL9584 | 150 | X |  |
| Depressariidae | *Agonopterix liturosa* | Haworth, 1811 | BOLD:AAE7191 | 150 | X |  |
| Elachistidae | *Elachista occidentalis* | Frey, 1882 | BOLD:AAD9988 | 150 | X |  |
| Noctuidae | *Mniotype adusta* | (Esper, 1790) | BOLD:AAD5982 | 150 | X |  |
| Tineidae | *Nemapogon cloacella* | (Haworth, 1828) | BOLD:AAC5133 | 150 | X |  |
| Noctuidae | *Craniophora ligustri* | Schiffermüller, 1776 | BOLD:AAB6108 | 150 | X |  |
| Crambidae | *Nomophila noctuella* | Schiffermüller, 1775 | BOLD:AAA7880 | 150 | X |  |
| Depressariidae | *Depressaria sordidatella* | Tengström, 1848 | BOLD:AAD6065 | 140 | X | X |
| Tortricidae | *Acleris rufana* | Denis & Schiffermüller, 1775 | BOLD:ACE5978 | 70 | X |  |
| Noctuidae | *Xestia ochreago* | Hübner, 1809 | BOLD:AAJ9109 | 70 | X | X |
| Depressariidae | *Agonopterix angelicella* | (Hübner, 1813) | BOLD:AAE3381 | 70 | X | X |
| Noctuidae | *Photedes minima* | Haworth, 1809 | BOLD:AAC7939 | 70 | X |  |
| Noctuidae | *Papestra biren* | Goeze, 1781 | BOLD:AAA9849 | 70 | X | X |
|  |  |  |  |  |  |  |
|  | Species identification – |  | BIN | Score | Sorted | Combined |
|  | **Coleoptera** |  |  |  |  |  |
| Melandryidae | *Orchesia minor* | *Walker 1837* | BOLD:AAO4777 | 1360 | X | X |
| Curculionidae | *Phyllobius arborator* | *Schilsky, J. in Küster , 1908* | BOLD:AAY1588 | 1060 | X | X |
| Lycidae | *Platycis minuta* | *(Fabricius, 1787)* | BOLD:ACB2638 | 1060 | X | X |
| Silphidae | *Nicrophorus vespilloides* | *Herbst, 1783* | BOLD:AAF3432 | 1020 | X | X |
| Cerambycidae | *Corymbia rubra* | *(Linnaeus, 1758)* | BOLD:AAI8975 | 1020 | X | X |
| Scraptiidae | *Anaspis rufilabris* | *(Gyllenhal, 1827)* | BOLD:AAN3436 | 1020 | X | X |
| Staphylinidae | *Lordithon exoletus* | *(Erichson 1839)* | BOLD:ACA7840 | 1020 | X | X |
| Chrysomelidae | *Gonioctena quinquepunctata* | *(Fabricius, 1787)* | BOLD:ABA7194 | 750 | X | X |
| Staphylinidae | *Lordithon lunulatus* | *(Linnaeus 1760)* | BOLD:AAO1349 | 730 | X | X |
| Leiodidae | *Sciodrepoides watsoni* | *(Spence 1815)* | BOLD:AAI0827 | 680 | X | X |
| Staphylinidae | *Aleochara curtula* | *(Goeze, 1777)* | BOLD:AAJ2741 | 680 | X | X |
| Sphindidae | *Aspidiphorus orbiculatus* | *(Gyllenhal 1808)* | BOLD:AAJ9399 | 680 | X | X |
| Staphylinidae | *Tachinus laticollis* | *Gravenhorst,1802* | BOLD:AAM9923 | 580 | X | X |
| Melyridae | *Dasytes plumbeus* | *(Müller, 1776)* | BOLD:AAO4702 | 580 | X | X |
| Chrysomelidae | *Longitarsus testaceus* | *(Melsheimer, 1847)* | BOLD:AAP8332 | 580 | X | X |
| Staphylinidae | *Lordithon thoracicus* | *(Fabricius 1777)* | BOLD:ABA9749 | 580 | X | X |
| Staphylinidae | *Atheta nigritula* | *(Gravenhorst, 1802)* | BOLD:ACL0069 | 580 | X | X |
| Staphylinidae | *Ontholestes murinus* | *Linnaeus,1758* | BOLD:AAP6128 | 490 | X | X |
| Leiodidae | *Choleva glauca* | *Britten, 1918* | BOLD:ACB7921 | 480 | X | X |
| Staphylinidae | *Lordithon bimaculatus* | *(Couper, 1865)* | BOLD:ABW5002 | 390 | X | X |
| Chrysomelidae | *Phyllotreta undulata* | *(Kutschera, 1860)* | BOLD:AAN1718 | 340 | X | X |
| Staphylinidae | *Euplectus kirbyi* | *Denny, 1825* | BOLD:AAO0162 | 340 | X | X |
| Scirtidae | *Elodes minuta* | *(Linnaeus, 1767)* | BOLD:ACG0990 | 340 | X | X |
| Staphylinidae | *Atheta castanoptera* | *(Mannerheim, 1830)* | BOLD:ABW6933 | 340 | X | X |
| Dytiscidae | *Laccophilus hyalinus* | *(De Geer, 1774)* | BOLD:ABX8802 | 340 | X |  |
| Staphylinidae | *Microscydmus minimus* | *(Chaudoir 1845)* | BOLD:ACC4328 | 340 | X | X |
| Cerambycidae | *Stictoleptura rubra* | *(Linnaeus, 1758)* | BOLD:AAJ8299 | 310 | X | X |
| Staphylinidae | *Agaricochara latissima* | *(Stephens, 1832)* | BOLD:ABA4424 | 300 | X | X |
| Leiodidae | *Leiodes polita* | *(Marsham, 1802)* | BOLD:AAO1258 | 240 | X | X |
| Latridiidae | *Enicmus rugosus* | *(Herbst, 1793)* | BOLD:ABX9520 | 240 | X | X |
| Chrysomelidae | *Altica opacifrons* | *(Lindberg, 1938)* | BOLD:AAQ1303 | 220 | X |  |
| Chrysomelidae | *Gonioctena intermedia* | *Helliesen, 1913* | BOLD:ACA9318 | 150 | X |  |
| Staphylinidae | *Gymnusa brevicollis* | *(Paykull, 1800)* | BOLD:AAY2458 | 150 | X | X |
|  |  |  |  |  |  |  |
|  |  |  |  |  |  |  |
| Family | Species Identification – |  | BIN | Score | Sorted | Combined |
|  | **Diptera** |  |  |  |  |  |
| Sciaridae | *Bradysia placida* | *(Winnertz, 1867)* | BOLD:ACR4350 | 1360 | X | X |
| Mycetophilidae | *Acnemia nitidicollis* | *(Meigen, 1818)* | BOLD:AAY5351 | 340 | X | X |
| Agromyzidae | Agromyzidae | Fallén, 1810 | BOLD:ACN4131 | 680 | X | X |
| Agromyzidae | Agromyzidae | Fallén, 1810 | BOLD:AAM6329 | 310 | X | X |
| Mycetophilidae | *Allodia truncata* | *Edwards, 1921* | BOLD:ABA7177 | 240 | X | X |
| Phoridae | *Anevrina thoracica* | *(Meigen, 1804)* | BOLD:ABA6986 | 340 | X |  |
| Anthomyiidae | Anthomyiidae | Latreille, 1829 | BOLD:AAG1783 | 750 | X | X |
| Anthomyiidae | Anthomyiidae | Latreille, 1829 | BOLD:AAG2463 | 580 | X | X |
| Anthomyiidae | Anthomyiidae | Latreille, 1829 | BOLD:AAN5494 | 70 | X |  |
| Chloropidae | *Aphanotrigonum nigripes* | *(Zetterstedt, 1848)* | BOLD:ACB9088 | 680 | X | X |
| Syrphidae | *Arctophila superbiens* | *(Muller, 1776)* | BOLD:AAZ1493 | 680 | X |  |
| Bibionidae | Bibionidae | Fleming, 1821 | BOLD:ACI4790 | 1020 | X | X |
| Sciaridae | *Bradysia affinis* | *(Zetterstedt, 1838)* | BOLD:ACC5704 | 1020 | X | X |
| Sciaridae | *Bradysia cinerascens* | *(Grzegorzek, 1884)* | BOLD:ACD6818 | 1020 | X | X |
| Sciaridae | *Bradysia reflexa* | *Tuomikoski 1960* | BOLD:ACP9275 | 300 | X |  |
| Sciaridae | *Bradysia* sp. | *Winnertz, 1867* | BOLD:ACD9543 | 340 | X |  |
| Sciaridae | *Bradysia* sp. | *Winnertz, 1868* | BOLD:ACG3678 | 240 | X |  |
| Sciaridae | *Bradysia trivittata* | *(Staeger, 1840)* | BOLD:ACE4654 | 340 | X |  |
| Mycetophilidae | *Brevicornu sericoma* | *(Meigen, 1830)* | BOLD:ABA1564 | 340 | X | X |
| Chironomidae | *Bryophaenocladius vernalis* | *(Goetghebuer, 1921)* | BOLD:AAP6930 | 340 | X | X |
| Chironomidae | *Bryophaenocladius ictericus* | *(Meigen, 1830)* | BOLD:AAM6273 | 340 | X | X |
| Cecidomyiidae | Cecidomyiidae | Newman, 1834 | BOLD:ACI7021 | 1020 | X | X |
| Cecidomyiidae | Cecidomyiidae | Newman, 1834 | BOLD:ACJ3318 | 1020 | X | X |
| Cecidomyiidae | Cecidomyiidae | Newman, 1834 | BOLD:ACF7865 | 920 | X | X |
| Cecidomyiidae | Cecidomyiidae | Newman, 1834 | BOLD:ACG7620 | 920 | X | X |
| Cecidomyiidae | Cecidomyiidae | Newman, 1834 | BOLD:ACH2823 | 830 | X |  |
| Cecidomyiidae | Cecidomyiidae | Newman, 1834 | BOLD:AAH3617 | 820 | X | X |
| Cecidomyiidae | Cecidomyiidae | Newman, 1834 | BOLD:ACI6441 | 820 | X |  |
| Cecidomyiidae | Cecidomyiidae | Newman, 1834 | BOLD:AAZ5610 | 680 | X | X |
| Cecidomyiidae | Cecidomyiidae | Newman, 1834 | BOLD:ACB9937 | 680 | X |  |
| Cecidomyiidae | Cecidomyiidae | Newman, 1834 | BOLD:ACC5872 | 680 | X |  |
| Cecidomyiidae | Cecidomyiidae | Newman, 1834 | BOLD:ACC6403 | 680 | X | X |
| Cecidomyiidae | Cecidomyiidae | Newman, 1834 | BOLD:ACG4666 | 680 | X |  |
| Cecidomyiidae | Cecidomyiidae | Newman, 1834 | BOLD:ACI5477 | 680 | X | X |
| Cecidomyiidae | Cecidomyiidae | Newman, 1834 | BOLD:ACJ3214 | 680 | X | X |
| Cecidomyiidae | Cecidomyiidae | Newman, 1834 | BOLD:ACP6507 | 680 | X |  |
| Cecidomyiidae | Cecidomyiidae | Newman, 1834 | BOLD:ACQ8906 | 680 | X |  |
| Cecidomyiidae | Cecidomyiidae | Newman, 1834 | BOLD:AAH3664 | 580 | X | X |
| Cecidomyiidae | Cecidomyiidae | Newman, 1834 | BOLD:ACE8241 | 580 | X |  |
| Cecidomyiidae | Cecidomyiidae | Newman, 1834 | BOLD:ACF9580 | 580 | X | X |
| Cecidomyiidae | Cecidomyiidae | Newman, 1834 | BOLD:ACG5399 | 580 | X | X |
| Cecidomyiidae | Cecidomyiidae | Newman, 1834 | BOLD:ACH3004 | 580 | X | X |
| Cecidomyiidae | Cecidomyiidae | Newman, 1834 | BOLD:ACI4027 | 580 | X |  |
| Cecidomyiidae | Cecidomyiidae | Newman, 1834 | BOLD:ACJ3537 | 580 | X |  |
| Cecidomyiidae | Cecidomyiidae | Newman, 1834 | BOLD:ABX7961 | 560 | X |  |
| Cecidomyiidae | Cecidomyiidae | Newman, 1834 | BOLD:ACF0010 | 490 | X |  |
| Cecidomyiidae | Cecidomyiidae | Newman, 1834 | BOLD:ACJ1102 | 490 | X | X |
| Cecidomyiidae | Cecidomyiidae | Newman, 1834 | BOLD:ACB9805 | 410 | X | X |
| Cecidomyiidae | Cecidomyiidae | Newman, 1834 | BOLD:AAH3623 | 340 | X |  |
| Cecidomyiidae | Cecidomyiidae | Newman, 1834 | BOLD:AAN5190 | 340 | X |  |
| Cecidomyiidae | Cecidomyiidae | Newman, 1834 | BOLD:ABV0466 | 340 | X |  |
| Cecidomyiidae | Cecidomyiidae | Newman, 1834 | BOLD:ACC0326 | 340 | X |  |
| Cecidomyiidae | Cecidomyiidae | Newman, 1834 | BOLD:ACC0880 | 340 | X |  |
| Cecidomyiidae | Cecidomyiidae | Newman, 1834 | BOLD:ACC1610 | 340 | X |  |
| Cecidomyiidae | Cecidomyiidae | Newman, 1834 | BOLD:ACF6114 | 340 | X | X |
| Cecidomyiidae | Cecidomyiidae | Newman, 1834 | BOLD:ACF6805 | 340 | X | X |
| Cecidomyiidae | Cecidomyiidae | Newman, 1834 | BOLD:ACF7864 | 340 | X |  |
| Cecidomyiidae | Cecidomyiidae | Newman, 1834 | BOLD:ACF8502 | 340 | X |  |
| Cecidomyiidae | Cecidomyiidae | Newman, 1834 | BOLD:ACF9583 | 340 | X | X |
| Cecidomyiidae | Cecidomyiidae | Newman, 1834 | BOLD:ACG4001 | 340 | X | X |
| Cecidomyiidae | Cecidomyiidae | Newman, 1834 | BOLD:ACG5730 | 340 | X |  |
| Cecidomyiidae | Cecidomyiidae | Newman, 1834 | BOLD:ACG7065 | 340 | X | X |
| Cecidomyiidae | Cecidomyiidae | Newman, 1834 | BOLD:ACJ1071 | 340 | X |  |
| Cecidomyiidae | Cecidomyiidae | Newman, 1834 | BOLD:ACJ3288 | 340 | X | X |
| Cecidomyiidae | Cecidomyiidae | Newman, 1834 | BOLD:ACK5108 | 340 | X | X |
| Cecidomyiidae | Cecidomyiidae | Newman, 1834 | BOLD:ACC1592 | 340 | X | X |
| Cecidomyiidae | Cecidomyiidae | Newman, 1834 | BOLD:ACC5341 | 340 | X |  |
| Cecidomyiidae | Cecidomyiidae | Newman, 1834 | BOLD:ACJ3384 | 340 | X |  |
| Cecidomyiidae | Cecidomyiidae | Newman, 1834 | BOLD:ACJ5431 | 340 | X |  |
| Cecidomyiidae | Cecidomyiidae | Newman, 1834 | BOLD:ACO0678 | 340 | X | X |
| Cecidomyiidae | Cecidomyiidae | Newman, 1834 | BOLD:ACQ8578 | 340 | X |  |
| Cecidomyiidae | Cecidomyiidae | Newman, 1834 | BOLD:ACQ8768 | 340 | X |  |
| Cecidomyiidae | Cecidomyiidae | Newman, 1834 | BOLD:ACQ9635 | 340 | X |  |
| Cecidomyiidae | Cecidomyiidae | Newman, 1834 | BOLD:ACR0427 | 340 | X |  |
| Cecidomyiidae | Cecidomyiidae | Newman, 1834 | BOLD:ACE2219 | 310 | X | X |
| Cecidomyiidae | Cecidomyiidae | Newman, 1834 | BOLD:ACB9928 | 240 | X |  |
| Cecidomyiidae | Cecidomyiidae | Newman, 1834 | BOLD:ACC1372 | 240 | X |  |
| Cecidomyiidae | Cecidomyiidae | Newman, 1834 | BOLD:ACC1591 | 240 | X | X |
| Cecidomyiidae | Cecidomyiidae | Newman, 1834 | BOLD:ACE6038 | 240 | X |  |
| Cecidomyiidae | Cecidomyiidae | Newman, 1834 | BOLD:ACF7975 | 240 | X | X |
| Cecidomyiidae | Cecidomyiidae | Newman, 1834 | BOLD:ACF8064 | 240 | X | X |
| Cecidomyiidae | Cecidomyiidae | Newman, 1834 | BOLD:ACF8067 | 240 | X |  |
| Cecidomyiidae | Cecidomyiidae | Newman, 1834 | BOLD:ACF9582 | 240 | X | X |
| Cecidomyiidae | Cecidomyiidae | Newman, 1834 | BOLD:ACG3481 | 240 | X | X |
| Cecidomyiidae | Cecidomyiidae | Newman, 1834 | BOLD:ACG3614 | 240 | X |  |
| Cecidomyiidae | Cecidomyiidae | Newman, 1834 | BOLD:ACG3846 | 240 | X |  |
| Cecidomyiidae | Cecidomyiidae | Newman, 1834 | BOLD:ACG4166 | 240 | X |  |
| Cecidomyiidae | Cecidomyiidae | Newman, 1834 | BOLD:ACG4458 | 240 | X |  |
| Cecidomyiidae | Cecidomyiidae | Newman, 1834 | BOLD:ACI8025 | 240 | X |  |
| Cecidomyiidae | Cecidomyiidae | Newman, 1834 | BOLD:ACJ3134 | 240 | X |  |
| Cecidomyiidae | Cecidomyiidae | Newman, 1834 | BOLD:ACJ3535 | 240 | X | X |
| Cecidomyiidae | Cecidomyiidae | Newman, 1834 | BOLD:ACM1467 | 240 | X |  |
| Cecidomyiidae | Cecidomyiidae | Newman, 1834 | BOLD:ACP5708 | 240 | X |  |
| Cecidomyiidae | Cecidomyiidae | Newman, 1834 | BOLD:ACR0633 | 240 | X |  |
| Cecidomyiidae | Cecidomyiidae | Newman, 1834 | BOLD:ACR5110 | 240 | X | X |
| Cecidomyiidae | Cecidomyiidae | Newman, 1834 | BOLD:ABV0448 | 150 | X | X |
| Cecidomyiidae | Cecidomyiidae | Newman, 1834 | BOLD:ABV1375 | 150 | X | X |
| Cecidomyiidae | Cecidomyiidae | Newman, 1834 | BOLD:ACE2294 | 150 | X | X |
| Cecidomyiidae | Cecidomyiidae | Newman, 1834 | BOLD:ACF9581 | 150 | X |  |
| Cecidomyiidae | Cecidomyiidae | Newman, 1834 | BOLD:ACG3551 | 150 | X | X |
| Cecidomyiidae | Cecidomyiidae | Newman, 1834 | BOLD:ACG3612 | 150 | X |  |
| Cecidomyiidae | Cecidomyiidae | Newman, 1834 | BOLD:ACG6315 | 150 | X |  |
| Cecidomyiidae | Cecidomyiidae | Newman, 1834 | BOLD:ACG7075 | 150 | X |  |
| Cecidomyiidae | Cecidomyiidae | Newman, 1834 | BOLD:ACJ3273 | 150 | X |  |
| Cecidomyiidae | Cecidomyiidae | Newman, 1834 | BOLD:ACQ9964 | 150 | X |  |
| Cecidomyiidae | Cecidomyiidae | Newman, 1834 | BOLD:ABX9178 | 70 | X |  |
| Cecidomyiidae | Cecidomyiidae | Newman, 1834 | BOLD:ACG4023 | 70 | X |  |
| Cecidomyiidae | Cecidomyiinae | Newman, 1834 | BOLD:ACQ9064 | 340 | X |  |
| Ceratopogonidae | Ceratopogonidae | Newman, 1834 | BOLD:ACF7463 | 390 | X |  |
| Ceratopogonidae | Ceratopogonidae | Newman, 1834 | BOLD:ACE1944 | 150 | X |  |
| Limoniidae | *Cheilotrichia cinerascens* | *(Meigen, 1804)* | BOLD:ABV4106 | 680 | X | X |
| Chloropidae | Chloropidae | Rondani, 1856 | BOLD:ACQ8508 | 680 | X | X |
| Dolichopodidae | *Chrysotus neglectus* | *(Wiedemann, 1817)* | BOLD:ACR4592 | 1260 | X | X |
| Dolichopodidae | *Chrysotus* sp. | *Meigen, 1824* | BOLD:ACP9364 | 240 | X | X |
| Sciaridae | *Claustropyga abblanda* | *(Freeman, 1983)* | BOLD:ABA6493 | 340 | X |  |
| Empididae | *Clinocera wesmaeli* | *(Macquart, 1835)* | BOLD:ACJ7323 | 340 | X |  |
| Mycetophilidae | *Coelophthinia thoracica* | *(Winnertz, 1863)* | BOLD:ACJ0721 | 240 | X | X |
| Muscidae | *Coenosia agromyzina* | *(Fallen, 1825)* | BOLD:ACR3782 | 70 | X |  |
| Muscidae | *Coenosia lineatipes* | *(Zetterstedt 1845)* | BOLD:ACG3538 | 240 | X | X |
| Mycetophilidae | *Cordyla brevicornis* | *(Staeger, 1840)* | BOLD:AAY5643 | 680 | X |  |
| Mycetophilidae | *Cordyla brevicornis* | *(Staeger, 1840)* | BOLD:AAY7669 | 680 | X |  |
| Mycetophilidae | *Cordyla fusca* | *Meigen, 1804* | BOLD:AAJ5766 | 240 | X | X |
| Sciaridae | *Corynoptera blanda* | *(Winnertz, 1867)* | BOLD:ABA5293 | 340 | X |  |
| Sciaridae | *Corynoptera boletiphaga* | *(Lengersdorf, 1940)* | BOLD:ACB9974 | 340 | X | X |
| Sciaridae | *Corynoptera forcipata* | *(Winnertz, 1867)* | BOLD:ACR4521 | 340 | X | X |
| Sciaridae | *Corynoptera membranigera* | *(Kieffer 1903)* | BOLD:ACG7087 | 340 | X | X |
| Sciaridae | *Corynoptera* sp. | *Winnertz, 1867* | BOLD:ACJ5436 | 920 | X | X |
| Sciaridae | *Corynoptera trepida* | *(Winnertz 1867)* | BOLD:ACC0330 | 480 | X | X |
| Sciaridae | *Cratyna vagabunda* | *(Winnertz, 1867)* | BOLD:ACK1466 | 340 | X | X |
| Diadocidiidae | *Diadocidia ferruginosa* | *(Meigen, 1830)* | BOLD:AAY7756 | 240 | X | X |
| Diadocidiidae | *Diadocidia spinosula* | *Tollet, 1948* | BOLD:ACE2644 | 680 | X | X |
| Tipulidae | *Dictenidia bimaculata* | *(Linnaeus, 1760)* | BOLD:ABU9173 | 580 | X | X |
| Phoridae | *Diplonevra funebris* | *(Meigen, 1830)* | BOLD:ACB3653 | 340 | X |  |
| Anthomyiidae | Diptera | Linnaeus, 1758 | BOLD:AAG2460 | 1020 | X | X |
| Muscidae | Diptera | Linnaeus, 1758 | BOLD:AAE0058 | 680 | X | X |
|  | Diptera | Linnaeus, 1758 | BOLD:ABA6492 | 580 | X | X |
|  | Diptera | Linnaeus, 1758 | BOLD:AAZ0264 | 460 | X | X |
| Mycetophilidae | Diptera | Linnaeus, 1758 | BOLD:AAG4873 | 300 | X | X |
| Mycetophilidae | Diptera | Linnaeus, 1758 | BOLD:AAI8810 | 220 | X | X |
| Sciaridae | *Dolichosciara nigrovittata* | *(Strobl, 1910)* | BOLD:ACG7846 | 340 | X | X |
| Hybotidae | *Drapetis parilis* | *Collin, 1926* | BOLD:ACE1676 | 580 | X | X |
| Drosophilidae | *Drosophila transversa* | *Fallen, 1823* | BOLD:ACB9860 | 680 | X |  |
| Tachinidae | *Eloceria delecta* | *(Meigen, 1824)* | BOLD:ACA9834 | 380 | X | X |
| Anthomyiidae | *Emmesomyia grisea* | *(Robineau-Desvoidy, 1830)* | BOLD:ACR4546 | 1020 | X | X |
| Empididae | *Empis* sp. | *Linnaeus, 1758* | BOLD:ACQ9954 | 920 | X | X |
| Ephydridae | Ephydridae | Zetterstedt, 1837 | BOLD:ACF1575 | 340 | X |  |
| Mycetophilidae | *Epicypta testata* | *Edwards, 1925* | BOLD:ACF9393 | 340 | X |  |
| Mycetophilidae | *Epicypta testata* | *Edwards, 1925* | BOLD:ACI4115 | 340 | X | X |
| Sciaridae | *Epidapus gracilis* | *(Walker, 1848)* | BOLD:ACF6656 | 340 | X | X |
| Sciaridae | *Epidapus microthorax* | *(Börner, 1903)* | BOLD:ACF7676 | 580 | X |  |
| Sciaridae | *Epidapus schillei* | *(Börner, 1903)* | BOLD:ACQ8624 | 580 | X |  |
| Syrphidae | *Episyrphus* sp. | *Matsumura & Adachi, 1917* | BOLD:ACF2414 | 1020 | X | X |
| Tachinidae | *Eriothrix rufomaculatus* | *(de Geer, 1776)* | BOLD:AAV7115 | 340 | X | X |
| Syrphidae | *Eristalis pertinax* | *(Scopoli, 1763)* | BOLD:AAQ3585 | 240 | X | X |
| Pipunculidae | *Eudorylas* sp. | *Aczel, 1940* | BOLD:ACE1834 | 1020 | X | X |
| Syrphidae | *Eumerus flavitarsis* | *Zetterstedt, 1843* | BOLD:AAQ1830 | 340 | X |  |
| Syrphidae | *Eupeodes lapponicus* | *(Zetterstedt, 1838)* | BOLD:AAB5561 | 1020 | X |  |
| Mycetophilidae | *Exechia fusca* | *(Meigen, 1804)* | BOLD:AAL5088 | 240 | X |  |
| Fanniidae | *Fannia polychaeta* | *(Stein, 1895)* | BOLD:ACQ8575 | 680 | X | X |
| Fanniidae | *Fannia polychaeta* | *(Stein, 1895)* | BOLD:ACD2937 | 650 | X | X |
| Fanniidae | Fanniidae | Townsend, 1935 | BOLD:AAG4620 | 240 | X | X |
| Syrphidae | *Ferdinandea ruficornis* | *(Fabricius, 1775)* | BOLD:AAQ4090 | 340 | X | X |
| Ceratopogonidae | *Forcipomyia alacris* | *(Winnertz, 1852)* | BOLD:ABW3964 | 340 | X | X |
| Ceratopogonidae | *Forcipomyia nigra* | *(Winnertz, 1852)* | BOLD:ABW3942 | 340 | X | X |
| Ceratopogonidae | *Forcipomyia* sp. | *Meigen, 1818* | BOLD:ACF8504 | 680 | X |  |
| Ceratopogonidae | *Forcipomyia* sp*. 4ES* | *Meigen, 1818* | BOLD:ACQ8860 | 540 | X | X |
| Ceratopogonidae | *Forcipomyia* sp*. 4ES* | *Meigen, 1818* | BOLD:ACQ8860 | 540 | X |  |
| Ceratopogonidae | *Forcipomyia* sp*. 4ES* | *Meigen, 1818* | BOLD:AAM6200 | 220 | X |  |
| Ceratopogonidae | *Forcipomyia* sp*. 6ES* | *Meigen, 1818* | BOLD:AAG6501 | 340 | X | X |
| Ceratopogonidae | *Forcipomyia squamigera* | *Kieffer & Thienemann, 1916* | BOLD:ACA4431 | 340 | X | X |
| Chloropidae | *Hapleginella laevifrons* | *(Loew, 1858)* | BOLD:ACD1355 | 150 | X |  |
| Heleomyzidae | Heleomyzidae | Bezzi, 1911 | BOLD:AAG0467 | 490 | X |  |
| Muscidae | *Helina impuncta* | *(Fallen, 1825)* | BOLD:AAX1493 | 940 | X | X |
| Heleomyzidae | *Heteromyza rotundicornis* | *(Zetterstedt, 1846)* | BOLD:ACD3128 | 720 | X | X |
| Hybotidae | *Hybos culiciformis* | *(Fabricius, 1775)* | BOLD:ACD8518 | 410 | X |  |
| Hybotidae | *Hybos grossipes* | *(Linnaeus, 1767)* | BOLD:ACC6150 | 240 | X | X |
| Hybotidae | Hybotidae | Fallén, 1816 | BOLD:AAH2937 | 340 | X | X |
| Hybotidae | Hybotidae | Fallén, 1816 | BOLD:ACB6879 | 340 | X |  |
| Hybotidae | Hybotidae | Fallén, 1816 | BOLD:ACG0645 | 340 | X |  |
| Muscidae | *Hydrotaea cyrtoneurina* | *(Zetterstedt, 1845)* | BOLD:AAX2553 | 1190 | X |  |
| Muscidae | *Hydrotaea irritans* | *(Fallen, 1823)* | BOLD:AAX2545 | 340 | X | X |
| Cecidomyiidae | Cecidomyiidae | Newman, 1834 | BOLD:ACR2287 | 150 | X |  |
| Keroplatidae | Keroplatidae | Rondani, 1856 | BOLD:ACC5977 | 820 | X | X |
| Keroplatidae | Keroplatidae | Rondani, 1856 | BOLD:ACF6392 | 150 | X |  |
| Chironomidae | *Krenopelopia* sp*.* | *Fittkau, 1962* | BOLD:AAI2213 | 1020 | X | X |
| Chironomidae | *Krenopelopia* sp*.* | *Fittkau, 1962* | BOLD:AAC9197 | 680 | X | X |
| Chironomidae | *Krenopelopia* sp. | *Fittkau, 1962* | BOLD:AAC9196 | 340 | X |  |
| Lauxaniidae | Lauxaniidae | Macquart, 1835 | BOLD:ABX3096 | 390 | X | X |
| Mycetophilidae | *Leia cylindrica* | *(Winnertz, 1863)* | BOLD:AAK8058 | 580 | X | X |
| Sciaridae | *Leptosciarella scutellata* | *(Staeger, 1840)* | BOLD:ACD1218 | 150 | X | X |
| Sciaridae | *Leptosciarella scutellata* | *(Staeger, 1840)* | BOLD:ACD6061 | 150 | X |  |
| Chironomidae | *Limnophyes habilis* | *(Walker, 1856)* | BOLD:AAJ2130 | 580 | X | X |
| Chironomidae | *Limnophyes minimus* | *(Meigen, 1818)* | BOLD:AAA8204 | 340 | X | X |
| Chironomidae | *Limnophyes natalensis* | *(Kieffer, 1914)* | BOLD:AAB7361 | 240 | X | X |
| Chironomidae | *Limnophyes pentaplastus* | *(Kieffer, 1921)* | BOLD:AAE6392 | 340 | X | X |
| Chironomidae | *Limnophyes* sp*. 8ES* | *Eaton, 1875* | BOLD:AAW1297 | 580 | X | X |
| Tachinidae | *Loewia* sp. | *Egger, 1856* | BOLD:ACP3825 | 340 | X | X |
| Tachinidae | *Lydina aenea* | *(Meigen, 1824)* | BOLD:ABY5280 | 340 | X | X |
| Dolichopodidae | *Medetera* sp*. SEB5* | *Fischer von Waldheim, 1819* | BOLD:ACA1124 | 340 | X | X |
| Phoridae | *Megaselia* sp. | *Rondani, 1856* | BOLD:AAG3264 | 340 | X | X |
| Phoridae | *Megaselia altifrons* | *(Wood, 1909)* | BOLD:ABA6996 | 340 | X | X |
| Phoridae | *Megaselia emarginata* | *(Wood, 1908)* | BOLD:ABA7003 | 340 | X | X |
| Phoridae | *Megaselia* sp. | *Rondani, 1856* | BOLD:ACG5397 | 480 | X | X |
| Phoridae | *Megaselia* sp. | *Rondani, 1856* | BOLD:ABU5529 | 340 | X | X |
| Phoridae | *Megaselia subtumida* | *(Wood, 1909)* | BOLD:ACD7791 | 240 | X | X |
| Syrphidae | *Melanostoma dubium* | *(Zetterstedt, 1838)* | BOLD:AAB2866 | 680 | X | X |
| Calliphoridae | *Melinda viridicyanea* | *(Robineau-Desvoidy, 1830)* | BOLD:ABX8360 | 890 | X | X |
| Limoniidae | *Metalimnobia quadrinotata* | *(Meigen, 1818)* | BOLD:ACF2132 | 390 | X | X |
| Sarcophagidae | *Metopia campestris* | *(Fallen, 1810)* | BOLD:AAG2427 | 1020 | X | X |
| Chironomidae | *Metriocnemus albolineatus* | *(Meigen, 1818)* | BOLD:AAC6643 | 680 | X | X |
| Limoniidae | *Molophilus* sp. | *Curtis, 1833* | BOLD:ACG3410 | 580 | X | X |
| Muscidae | *Muscina levida* | *Harris, 1780* | BOLD:AAB8817 | 680 | X | X |
| Mycetophilidae | *Mycetophila alea* | *Laffoon, 1965* | BOLD:AAG4931 | 150 | X | X |
| Mycetophilidae | *Mycetophila fungorum* | *(de Geer, 1776)* | BOLD:ABU7342 | 340 | X | X |
| Mycetophilidae | *Mycetophila luctuosa* | *Meigen, 1830* | BOLD:AAG3628 | 340 | X |  |
| Mycetophilidae | *Mycetophila marginata* | *Winnertz, 1863* | BOLD:AAY8339 | 340 | X |  |
| Mycetophilidae | *Mycetophila ornata* | *Stephens, 1829* | BOLD:ACJ6199 | 340 | X | X |
| Mycetophilidae | *Mycetophila* sp*. CJB3* | *Meigen, 1803* | BOLD:AAP8158 | 340 | X |  |
| Mycetophilidae | *Mycetophila* sp*. CJB7* | *Meigen, 1803* | BOLD:AAG4872 | 340 | X | X |
| Mycetophilidae | *Mycetophila stolida* | *Walker, 1856* | BOLD:AAY8337 | 580 | X |  |
| Mycetophilidae | Mycetophilidae | Newman, 1834 | BOLD:ACJ5307 | 340 | X |  |
| Mycetophilidae | Mycetophilidae | Newman, 1834 | BOLD:ACC1239 | 240 | X | X |
| Mycetophilidae | Mycetophilidae | Newman, 1834 | BOLD:ACI8126 | 240 | X | X |
| Mycetophilidae | Mycetophilidae | Newman, 1834 | BOLD:ACJ2631 | 240 | X | X |
| Mycetophilidae | Mycetophilidae | Newman, 1834 | BOLD:ACJ4960 | 240 | X | X |
| Mycetophilidae | Mycetophilidae | Newman, 1834 | BOLD:ACJ4957 | 150 | X |  |
| Mycetophilidae | Mycetophilidae | Newman, 1834 | BOLD:ACQ8258 | 680 | X | X |
| Mycetophilidae | *Mycomya marginata* | *(Meigen, 1818)* | BOLD:ACR1321 | 300 | X | X |
| Mycetophilidae | *Mycomya* sp. | *Rondani, 1856* | BOLD:ACC6515 | 240 | X | X |
| Mycetophilidae | *Mycomya winnertzi* | *(Dziedzicki, 1885)* | BOLD:AAY8344 | 340 | X |  |
| Muscidae | *Mydaea humeralis* | *Robineau-Desvoidy, 1830* | BOLD:ACD1934 | 240 | X | X |
| Muscidae | *Mydaea pseudonubila* | *Huckett, 1965* | BOLD:AAE0077 | 340 | X |  |
| Muscidae | *Mydaea urbana* | *(Meigen, 1826)* | BOLD:ACB9959 | 680 | X |  |
| Syrphidae | *Neoascia annexa* | *(Müller, 1776)* | BOLD:ACH5509 | 240 | X |  |
| Limoniidae | *Neolimonia dumetorum* | *(Meigen, 1804)* | BOLD:ABV5347 | 1020 | X | X |
| Milichiidae | *Neophyllomyza* sp. | *Melander, 1913* | BOLD:ACG4021 | 140 | X | X |
| Dolichopodidae | *Neurigona pallida* | *(Fallen, 1823)* | BOLD:ACB6721 | 340 | X |  |
| Dolichopodidae | *Neurigona quadrifasciata* | *(Fabricius, 1781)* | BOLD:ACB3226 | 580 | X | X |
| Agromyzidae | *Ophiomyia* sp. | *Braschnikov, 1897* | BOLD:ACP4138 | 150 | X |  |
| Mycetophilidae | *Palaeodocosia vittata* | *(Coquillett, 1901)* | BOLD:ACI0822 | 340 | X | X |
| Syrphidae | *Paragus tibialis* | *(Fallen, 1817)* | BOLD:ABZ4619 | 340 | X | X |
| Chironomidae | *Paraphaenocladius pseudirritus* | *Strenzke, 1950* | BOLD:AAC4194 | 580 | X | X |
| Anthomyiidae | *Pegomya solennis* | *(Meigen, 1826)* | BOLD:ACD8686 | 340 | X | X |
| Anthomyiidae | *Pegoplata* sp. | *Schnabl and Dziedzicki, 1911* | BOLD:ACC6060 | 1020 | X | X |
| Tachinidae | *Periscepsia spathulata* | *(Fallen, 1820)* | BOLD:ACE9427 | 820 | X | X |
| Muscidae | *Phaonia angelicae* | *(Scopoli, 1763)* | BOLD:ACI9785 | 480 | X | X |
| Muscidae | *Phaonia rufiventris* | *(Scopoli, 1763)* | BOLD:ABW1816 | 920 | X | X |
| Psychodidae | *Philosepedon* sp*. 1* | *Eaton, 1904* | BOLD:ABA0882 | 340 | X | X |
| Ephydridae | *Philygria vittipennis* | *(Zetterstedt, 1838)* | BOLD:AAP6349 | 340 | X |  |
| Phoridae | *Phora tincta* | *Schmitz, 1920* | BOLD:ABA7002 | 340 | X |  |
| Phoridae | Phoridae | Curtis, 1833 | BOLD:ACG4586 | 680 | X | X |
| Phoridae | Phoridae | Curtis, 1833 | BOLD:ACI9454 | 680 | X | X |
| Phoridae | Phoridae | Curtis, 1833 | BOLD:ACP3075 | 680 | X | X |
| Phoridae | Phoridae | Curtis, 1833 | BOLD:AAG3260 | 340 | X | X |
| Phoridae | Phoridae | Curtis, 1833 | BOLD:AAG3266 | 340 | X | X |
| Phoridae | Phoridae | Curtis, 1833 | BOLD:AAG7025 | 340 | X | X |
| Phoridae | Phoridae | Curtis, 1833 | BOLD:AAL9073 | 340 | X |  |
| Phoridae | Phoridae | Curtis, 1833 | BOLD:ACC0351 | 340 | X |  |
| Phoridae | Phoridae | Curtis, 1833 | BOLD:ACC0903 | 340 | X |  |
| Phoridae | Phoridae | Curtis, 1833 | BOLD:ACD9573 | 340 | X | X |
| Phoridae | Phoridae | Curtis, 1833 | BOLD:ACE2214 | 340 | X | X |
| Phoridae | Phoridae | Curtis, 1833 | BOLD:ACF6036 | 340 | X |  |
| Phoridae | Phoridae | Curtis, 1833 | BOLD:ACG6991 | 340 | X |  |
| Phoridae | Phoridae | Curtis, 1833 | BOLD:ACG8360 | 340 | X | X |
| Phoridae | Phoridae | Curtis, 1833 | BOLD:ACI4663 | 340 | X | X |
| Phoridae | Phoridae | Curtis, 1833 | BOLD:ACP1058 | 340 | X |  |
| Phoridae | Phoridae | Curtis, 1833 | BOLD:ACI8210 | 240 | X | X |
| Mycetophilidae | *Phronia nigricornis* | *(Zetterstedt, 1852)* | BOLD:ABA3510 | 240 | X |  |
| Mycetophilidae | *Phronia nitidiventris* | *(Wulp, 1858)* | BOLD:ACB2159 | 240 | X | X |
| Mycetophilidae | *Phronia strenua* | *Winnertz, 1863* | BOLD:ABA1330 | 1020 | X | X |
| Mycetophilidae | *Phthinia mira* | *(Ostroverkhova 1977)* | BOLD:ACF6717 | 680 | X | X |
| Sciaridae | *Phytosciara flavipes* | *(Meigen, 1804)* | BOLD:ACC5752 | 1020 | X | X |
| Tachinidae | *Platymya confusionis* | *(Sellers, 1943)* | BOLD:AAG2162 | 340 | X | X |
| Hybotidae | *Platypalpus pectoralis* | *(Fallen, 1815)* | BOLD:ACD2945 | 340 | X |  |
| Muscidae | *Polietes lardarius* | *(Fabricius, 1781)* | BOLD:AAY2766 | 340 | X | X |
| Calliphoridae | *Pollenia amentaria* | *(Scopoli, 1763)* | BOLD:ABV5497 | 680 | X | X |
| Chironomidae | *Pseudorthocladius* sp. | *Goetghebuer, 1943* | BOLD:ABV3504 | 150 | X | X |
| Chironomidae | *Pseudosmittia* sp. | *Goetghebeur, 1932* | BOLD:ACG7714 | 1020 | X | X |
| Psychodidae | *Psychoda gemina* | *(Eaton, 1904)* | BOLD:ACR3941 | 1020 | X | X |
| Psychodidae | *Psychoda grisescens* | *Tonnoir, 1922* | BOLD:AAM9202 | 340 | X |  |
| Psychodidae | *Psychoda lobata* | *Tonnoir, 1940* | BOLD:ABA0877 | 1020 | X | X |
| Psychodidae | *Psychoda phalaenoides* | *(Linnaeus, 1758)* | BOLD:AAF9317 | 680 | X | X |
| Psychodidae | *Psychoda satchelli* | *Quate 1955* | BOLD:ACG1924 | 680 | X | X |
| Psychodidae | *Psychoda* sp. | *Latreille, 1796* | BOLD:AAF9314 | 320 | X | X |
| Psychodidae | Psychodidae | Newman, 1834 | BOLD:ACG3216 | 580 | X | X |
| Rhagionidae | *Rhagio lineola* | *Fabricius, 1794* | BOLD:AAZ5903 | 680 | X |  |
| Rhagionidae | Rhagionidae | Latreille, 1802 | BOLD:ACJ2828 | 340 | X | X |
| Sarcophagidae | *Sarcophaga caerulescens* | *Zetterstedt, 1838* | BOLD:ABZ2577 | 820 | X | X |
| Sarcophagidae | *Sarcophaga carnaria* | *(Linnaeus, 1758)* | BOLD:AAX9423 | 820 | X | X |
| Sarcophagidae | *Sarcophaga depressifrons* | *Zetterstedt, 1845* | BOLD:ACB5889 | 240 | X | X |
| Sarcophagidae | *Sarcophaga incisilobata* | *Pandelle, 1896* | BOLD:ACB4675 | 680 | X | X |
| Sarcophagidae | *Sarcophaga subvicina* | *Baranov, 1937* | BOLD:AAG6743 | 220 | X | X |
| Sarcophagidae | Sarcophagidae | Macquart, 1834 | BOLD:AAG2171 | 820 | X | X |
| Sciaridae | *Scatopsciara atomaria* | *(Zetterstedt, 1851)* | BOLD:AAN6431 | 150 | X | X |
| Sciaridae | *Scatopsciara atomaria* | *(Zetterstedt, 1851)* | BOLD:ACB4497 | 70 | X |  |
| Sciaridae | *Scatopsciara edwardsi* | *Freeman, 1983* | BOLD:ACI6952 | 680 | X | X |
| Sciaridae | *Scatopsciara* sp. | *Edwards, 1927* | BOLD:AAH4004 | 70 | X |  |
| Scatopsidae | Scatopsidae | Newman, 1834 | BOLD:ACF6113 | 490 | X |  |
| Dolichopodidae | *Sciapus platypterus* | *(Fabricius, 1805)* | BOLD:ABU9505 | 340 | X | X |
| Sciaridae | Sciaridae | Billberg, 1820 | BOLD:AAH3983 | 920 | X | X |
| Sciaridae | Sciaridae | Billberg, 1820 | BOLD:ACA8369 | 680 | X |  |
| Sciaridae | Sciaridae | Billberg, 1820 | BOLD:AAG4963 | 340 | X |  |
| Sciaridae | Sciaridae | Billberg, 1820 | BOLD:AAV1366 | 240 | X |  |
| Mycetophilidae | *Sciophila* sp*. CJB1* | *Meigen, 1818* | BOLD:AAN8583 | 240 | X |  |
| Sepsidae | Sepsidae | Walker, 1833 | BOLD:AAG5640 | 340 | X | X |
| Syrphidae | *Sericomyia silentis* | *(Harris, 1776)* | BOLD:AAB1553 | 920 | X | X |
| Simuliidae | *Simulium vernum* | *(Macquart, 1826)* | BOLD:AAB8624 | 450 | X |  |
| Chironomidae | *Smittia* sp*. 8ES* | *Holmgren 1869* | BOLD:ACP4736 | 490 | X |  |
| Sphaeroceridae | *Sphaeroceridae* | *Macquart 1835* | BOLD:AAG7028 | 680 | X | X |
| Sphaeroceridae | Sphaeroceridae | Macquart, 1835 | BOLD:AAG7282 | 340 | X | X |
| Sphaeroceridae | Sphaeroceridae | Macquart, 1835 | BOLD:ABY1126 | 340 | X |  |
| Sphaeroceridae | Sphaeroceridae | Macquart, 1835 | BOLD:ACG3635 | 240 | X | X |
| Syrphidae | *Sphaerophoria* sp. | *(Linnaeus, 1758)* | BOLD:AAA7374 | 920 | X | X |
| Anisopodidae | *Sylvicola fenestralis* | *(Scopoli, 1763)* | BOLD:AAG1996 | 240 | X | X |
| Tachinidae | Tachinidae | Robineau-Desvoidy, 1830 | BOLD:AAG2108 | 340 | X |  |
| Tachinidae | Tachinidae | Robineau-Desvoidy, 1830 | BOLD:AAG2163 | 340 | X | X |
| Mycetophilidae | *Tetragoneura sylvatica* | *(Curtis, 1837)* | BOLD:AAG4900 | 580 | X | X |
| Muscidae | *Thricops cunctans* | *(Meigen, 1826)* | BOLD:AAK1780 | 580 | X | X |
| Muscidae | *Thricops diaphanus* | *(Wiedemann, 1817)* | BOLD:AAG1710 | 150 | X | X |
| Muscidae | *Thricops semicinereus* | *(Wiedemann, 1817)* | BOLD:ABV4563 | 680 | X | X |
| Tipulidae | *Tipula irrorata* | *Macquart, 1826* | BOLD:ACB7061 | 820 | X | X |
| Asilidae | *Tolmerus atricapillus* | *(Fallén, 1814)* | BOLD:ACI9053 | 340 | X | X |
| Pipunculidae | *Tomosvaryella kuthyi* | *Aczél, 1944* | BOLD:AAG1660 | 340 | X | X |
| Pipunculidae | *Tomosvaryella* sp. | *Aczél, 1939* | BOLD:ACD8703 | 240 | X |  |
| Hybotidae | *Trichina bilobata* | *Collin, 1926* | BOLD:ACB9912 | 680 | X | X |
| Sciaridae | *Trichosia acrotricha* | *Tuomikoski, 1960* | BOLD:ACF7707 | 340 | X |  |
| Rhinophoridae | *Tricogena rubricosa* | *(Meigen, 1824)* | BOLD:ACD2617 | 680 | X |  |
| Pediciidae | *Ula bolitophila* | *Loew, 1869* | BOLD:ABU5946 | 70 | X |  |
| Pediciidae | *Ula mixta* | *Stary, 1983* | BOLD:ABA7462 | 480 | X | X |
| Tachinidae | *Winthemia* sp. | *Robineau-Desvoidy, 1830* | BOLD:ABZ7811 | 340 | X |  |
| Syrphidae | *Xanthandrus comtus* | *(Harris, 1780)* | BOLD:AAZ6101 | 340 | X | X |
| Syrphidae | *Xylota segnis* | *(Linnaeus, 1758)* | BOLD:AAG4673 | 680 | X | X |
|  |  |  |  |  |  |  |
|  |  |  |  |  |  |  |
| Family | Species Identification – |  | BIN | Score | Sorted | Combined |
|  | **Hymenoptera** |  |  |  |  |  |
| Ichneumonidae | Ichneumonidae | Latreille, 1802 | BOLD:ACG3907 | 1260 | X |  |
| Ichneumonidae | *Acrotomus succinctus* | (Gravenhorst, 1829) | BOLD:AAH2187 | 340 | X | X |
| Ichneumonidae | *Agrothereutes abbreviatus* | (Fabricius, 1794) | BOLD:ACN8453 | 150 | X |  |
| Tenthredinidae | *Allantus rufocinctus* | (Retzius 1783) | BOLD:AAE6121 | 340 | X |  |
| Andrenidae | *Andrena helvola* | (Linnaeus, 1758) | BOLD:ABU9089 | 340 | X | X |
| Pompilidae | *Anoplius nigerrimus* | (Scopoli, 1763) | BOLD:AAN3329 | 920 | X | X |
| Ichneumonidae | *Aperileptus albipalpus* | (Gravenhorst, 1829) | BOLD:AAU9319 | 1020 | X | X |
| Ichneumonidae | *Aperileptus albipalpus* | (Gravenhorst, 1829) | BOLD:ABZ9967 | 460 | X | X |
| Ichneumonidae | *Aperileptus vanus* | Förster, 1871 | BOLD:ACL6838 | 340 | X |  |
| Ichneumonidae | *Aperileptus vanus* | Förster, 1871 | BOLD:ACG5754 | 240 | X |  |
| Apidae | *Apis mellifera* | Linnaeus, 1758 | BOLD:AAA2326 | 830 | X | X |
| Ichneumonidae | *Aptesis nigrocincta* | (Gravenhorst, 1815) | BOLD:ABX2295 | 340 | X |  |
| Ichneumonidae | *Astiphromma simplex* | (Thomson, 1886) | BOLD:ACF4110 | 240 | X | X |
| Tenthredinidae | *Athalia circularis circularis* | (Klug, 1815) | BOLD:AAC0157 | 580 | X |  |
| Tenthredinidae | *Athalia cordata* | Serville, 1823 | BOLD:AAP1621 | 1020 | X | X |
| Diapriidae | *Belyta depressa* | Thomson, 1859 | BOLD:ACH3381 | 340 | X |  |
| Apidae | *Bombus flavobarbatus* | Morawitz, 1883 | BOLD:AAC4378 | 680 | X |  |
| Apidae | *Bombus pratorum* | (Linnaeus, 1761) | BOLD:AAD4735 | 340 | X |  |
| Evaniidae | *Brachygaster minutus* | (Olivier, 1792) | BOLD:AAW7612 | 340 | X |  |
| Braconidae | Braconidae | Burmeister, 1829 | BOLD:AAH3139 | 680 | X | X |
| Braconidae | Braconidae | Burmeister, 1829 | BOLD:ACG7926 | 680 | X | X |
| Braconidae | Braconidae | Burmeister, 1829 | BOLD:AAI2846 | 340 | X |  |
| Braconidae | Braconidae | Burmeister, 1829 | BOLD:ACC4430 | 340 | X |  |
| Braconidae | Braconidae | Burmeister, 1829 | BOLD:ACJ7764 | 220 | X |  |
| Braconidae | Braconidae | Burmeister, 1829 | BOLD:ACN6851 | 150 | X |  |
| Braconidae | Braconidae | Burmeister, 1829 | BOLD:AAA3092 | 70 | X |  |
| Ichneumonidae | *Campoplex difformis* | (Gmelin, 1790) | BOLD:ACG4708 | 390 | X | X |
| Chrysididae | *Chrysis ignita* | (Linnaeus, 1758) | BOLD:AAU2329 | 70 | X |  |
| Chrysididae | *Chrysis mediata* | Linsenmaier, 1951 | BOLD:AAY6949 | 70 | X |  |
| Tenthredinidae | *Cladius compressicornis* | (Fabricius, 1804) | BOLD:ABX5569 | 340 | X |  |
| Tenthredinidae | *Cladius pectinicornis* | (Geoffroy, 1785) | BOLD:AAE2748 | 340 | X | X |
| Braconidae | *Cotesia* sp. *jft07* | Cameron, 1891 | BOLD:ABZ3751 | 70 | X |  |
| Ichneumonidae | *Cratichneumon viator* | (Scopoli, 1763) | BOLD:AAF2407 | 240 | X |  |
| Ichneumonidae | *Cryptopimpla errabunda* | (Gravenhorst, 1829) | BOLD:ACF8360 | 1020 | X |  |
| Ichneumonidae | *Cylloceria* sp*. SK-2009* | Schidte, 1838 | BOLD:AAI5705 | 240 | X |  |
| Ichneumonidae | *Deuteroxorides* sp*. SK-2009* | Viereck 1914 | BOLD:AAL1331 | 340 | X |  |
| Diapriidae | Diapriidae | Haliday, 1833 | BOLD:ACE0017 | 340 | X |  |
| Braconidae | *Dinotrema* sp. *jft01* | Foerster, 1862 | BOLD:AAA7636 | 70 | X |  |
| Ichneumonidae | *Diplazon laetatorius* | (Fabricius, 1781) | BOLD:AAD1879 | 680 | X | X |
| Ichneumonidae | *Diplazon laetatorius* | (Fabricius, 1781) | BOLD:AAD4214 | 240 | X |  |
| Ichneumonidae | *Diplazon scutatorius* | Teunissen, 1943 | BOLD:AAL2105 | 680 | X |  |
| Pompilidae | *Dipogon subintermedius* | (Magretti, 1886) | BOLD:AAN4111 | 820 | X |  |
| Ichneumonidae | *Dolichomitus imperator* | (Kriechbaumer, 1854) | BOLD:AAN3966 | 580 | X |  |
| Ichneumonidae | *Dyspetes luteomarginatus* | Habermehl, 1925 | BOLD:AAJ4814 | 680 | X |  |
| Eulophidae | Eulophidae | Westwood 1829 | BOLD:ACN3834 | 240 | X | X |
| Eupelmidae | Eupelmidae | Walker, 1833 | BOLD:AAZ0170 | 240 | X | X |
| Ichneumonidae | *Exephanes ischioxanthus* | (Gravenhorst, 1829) | BOLD:ACC3068 | 1020 | X | X |
| Ichneumonidae | *Exochus flavomarginatus* | Holmgren, 1856 | BOLD:ACI4522 | 340 | X |  |
| Figitidae | Figitidae | Thomson, 1862 | BOLD:AAU8589 | 340 | X | X |
| Formicidae | *Formica cinerea* | Mayr, 1853 | BOLD:AAA4978 | 920 | X |  |
| Halictidae | *Halictus rubicundus* | (Christ, 1791) | BOLD:AAA3534 | 240 | X |  |
| Ichneumonidae | *Helictes erythrostoma* | (Gmelin, 1790) | BOLD:AAY2005 | 290 | X | X |
| Ichneumonidae | Ichneumonidae | Latreille, 1802 | BOLD:ACR4592 | 1260 | X | X |
| Ichneumonidae | Ichneumonidae | Latreille, 1802 | BOLD:AAH1536 | 1020 | X | X |
| Ichneumonidae | Ichneumonidae | Latreille, 1802 | BOLD:ACH1883 | 1020 | X | X |
| Ichneumonidae | Ichneumonidae | Latreille, 1802 | BOLD:AAN8654 | 680 | X |  |
| Ichneumonidae | Ichneumonidae | Latreille, 1802 | BOLD:ACL7538 | 680 | X | X |
| Ichneumonidae | Ichneumonidae | Latreille, 1802 | BOLD:ACQ8576 | 680 | X | X |
| Ichneumonidae | Ichneumonidae | Latreille, 1802 | BOLD:ACE6827 | 680 | X | X |
| Ichneumonidae | Ichneumonidae | Latreille, 1802 | BOLD:ACC6524 | 580 | X | X |
| Ichneumonidae | Ichneumonidae | Latreille, 1802 | BOLD:ACC4370 | 480 | X | X |
| Ichneumonidae | Ichneumonidae | Latreille, 1802 | BOLD:ACC4770 | 340 | X | X |
| Ichneumonidae | Ichneumonidae | Latreille, 1802 | BOLD:ACI4374 | 340 | X | X |
| Ichneumonidae | Ichneumonidae | Latreille, 1802 | BOLD:ACP6077 | 340 | X |  |
| Ichneumonidae | Ichneumonidae | Latreille, 1802 | BOLD:ACQ9363 | 340 | X |  |
| Ichneumonidae | Ichneumonidae | Latreille, 1802 | BOLD:ACG7703 | 310 | X |  |
| Ichneumonidae | Ichneumonidae | Latreille, 1802 | BOLD:AAD5194 | 240 | X | X |
| Ichneumonidae | Ichneumonidae | Latreille, 1802 | BOLD:AAD5318 | 240 | X | X |
| Ichneumonidae | Ichneumonidae | Latreille, 1802 | BOLD:ACJ3006 | 70 | X |  |
| Ichneumonidae | Ichneumonidae | Latreille, 1802 | BOLD:ACJ3381 | 70 | X |  |
| Halictidae | *Lasioglossum fulvicorne* | (Kirby, 1802) | BOLD:AAY5432 | 150 | X |  |
| Halictidae | *Lasioglossum leucopus* | (Kirby, 1802) | BOLD:AAF3848 | 240 | X | X |
| Formicidae | *Lasius umbratus* | (Nylander, 1846) | BOLD:AAE1554 | 240 | X |  |
| Ichneumonidae | *Leipaulus* sp. *jft01* | Townes, 1945 | BOLD:AAX5466 | 240 | X |  |
| Ichneumonidae | *Lissonota coracina* | (Gmelin, 1790) | BOLD:ABU7646 | 70 | X |  |
| Megachilidae | *Megachile willughbiella* | (Kirby, 1802) | BOLD:ABZ3482 | 450 | X |  |
| Ichneumonidae | *Megastylus flavopictus* | (Gravenhorst, 1829) | BOLD:ACF9216 | 340 | X | X |
| Ichneumonidae | *Megastylus orbitator* | Schiodte, 1838 | BOLD:AAK5985 | 680 | X | X |
| Ichneumonidae | *Megastylus orbitator* | Schiodte, 1838 | BOLD:ABX6011 | 340 | X |  |
| Ichneumonidae | *Mevesia alternans* | (Wesmael, 1845) | BOLD:ACJ7824 | 340 | X |  |
| Braconidae | *Microgaster* sp. *jft10* | Bocquet-Védrine, 1967 | BOLD:ACE8790 | 340 | X |  |
| Braconidae | *Microplitis varicolor* | Viereck, 1917 | BOLD:AAA2408 | 650 | X | X |
| Tenthredinidae | *Nematus myosotidis* | (Fabricius, 1804) | BOLD:AAM4920 | 240 | X |  |
| Chrysididae | *Omalus aeneus* | (Fabricius,1787) | BOLD:ACC4462 | 680 | X |  |
| Ichneumonidae | *Orthocentrus* sp. *jft12* | Gravenhorst, 1829 | BOLD:AAC3876 | 820 | X | X |
| Ichneumonidae | *Pimpla contemplator* | (Müller, 1776) | BOLD:AAH9350 | 1020 | X | X |
| Ichneumonidae | *Pimpla contemplator* | (Müller, 1776) | BOLD:AAH9349 | 480 | X |  |
| Ichneumonidae | *Pimpla turionellae* | (Linnaeus, 1758) | BOLD:AAN3703 | 340 | X |  |
| Platygastridae | Platygastridae | Halliday, 1833 | BOLD:ACF7644 | 340 | X |  |
| Platygastridae | Platygastridae | Halliday, 1833 | BOLD:ACI4525 | 240 | X |  |
| Ichneumonidae | *Platylabus odiosus* | Perkins, 1953 | BOLD:ACI7609 | 70 | X |  |
| Ichneumonidae | *Plectiscus impurator* | Gravenhorst, 1829 | BOLD:ABU9109 | 680 | X |  |
| Ichneumonidae | *Polyblastus wahlbergi* | Holmgren, 1857 | BOLD:ACC0515 | 680 | X | X |
| Ichneumonidae | *Polysphincta boops* | Tschek, 1869 | BOLD:AAZ9003 | 150 | X |  |
| Pompilidae | *Priocnemis hyalinata* | (Fabricius, 1793) | BOLD:AAN4192 | 580 | X | X |
| Ichneumonidae | *Proclitus praetor* | (Haliday, 1838) | BOLD:ACE4931 | 580 | X | X |
| Cynipidae | *Synergus pallicornis* | Hartig, 1841 | BOLD:ACB7147 | 340 | X | X |
| Ichneumonidae | *Syrphoctonus tarsatorius* | (Panzer, 1809) | BOLD:AAK1321 | 1020 | X | X |
| Tenthredinidae | *Tenthredo frauenfeldii* | Giraud, 1857 | BOLD:ABU5889 | 340 | X |  |
| Ichneumonidae | *Thaumatogelis sylvicola* | (Forster, 1850) | BOLD:ACR1440 | 580 | X |  |
| Ichneumonidae | *Thaumatogelis sylvicola* | (Forster, 1850) | BOLD:ABU5806 | 70 | X |  |
| Crabronidae | *Trypoxylon minus* | Beaumont, 1945 | BOLD:AAF5985 | 340 | X | X |
| Ichneumonidae | *Tycherus bellicornis* | (Wesmael, 1845) | BOLD:AAN3733 | 70 | X |  |
