# Supplementary material for: Species Identification in Malaise Trap Samples by DNA Barcoding Based on NGS Technologies and a Scoring Matrix
Source: PLoS One. 2016 May 18;11(5):e0155497. doi: 10.1371/journal.pone.0155497 (PMC4871420; doi:10.1371/journal.pone.0155497)
Supplement: S2 Table — (DOCX) [file pone.0155497.s004.docx]

**S2 Table .** **List of all families per insect order studied and number of family representatives detected for each order.**

| Coleptera | |
| --- | --- |
|  |  |
| Family | Number of representative species |
| Cerambycidae | 2 |
| Chrysomelidae | 5 |
| Curculionidae | 1 |
| Dytiscidae | 1 |
| Latridiidae | 1 |
| Leiodidae | 3 |
| Lycidae | 1 |
| Melandryidae | 1 |
| Melyridae | 1 |
| Scirtidae | 1 |
| Scraptiidae | 1 |
| Silphidae | 1 |
| Sphindidae | 1 |
| Staphylinidae | 13 |

| Lepidoptera | |
| --- | --- |
|  |  |
| Family | Number of representative species |
| Argyresthiidae | 1 |
| Crambidae | 4 |
| Depressariidae | 5 |
| Elachistidae | 2 |
| Erebidae | 1 |
| Geometridae | 5 |
| Gracillariidae | 1 |
| Hepialidae | 1 |
| Nepticulidae | 1 |
| Noctuidae | 9 |
| Nymphalidae | 2 |
| Pieridae | 1 |
| Pyralidae | 2 |
| Tineidae | 1 |
| Tortricidae | 4 |

| Hymenoptera | |
| --- | --- |
|  |  |
| Family | Number of representative species |
| Andrenidae | 1 |
| Apidae | 3 |
| Braconidae | 11 |
| Chrysididae | 3 |
| Crabronidae | 1 |
| Cynipidae | 1 |
| Diapriidae | 2 |
| Eulophidae | 1 |
| Eupelmidae | 1 |
| Evaniidae | 1 |
| Figitidae | 1 |
| Formicidae | 2 |
| Halictidae | 3 |
| Ichneumonidae | 59 |
| Megachilidae | 1 |
| Platygastridae | 2 |
| Pompilidae | 3 |
| Tenthredinidae | 7 |

| Diptera | |
| --- | --- |
|  |  |
| Family | Number of representative species |
| Agromyzidae | 3 |
| Anisopodidae | 1 |
| Anthomyiidae | 7 |
| Asilidae | 1 |
| Bibionidae | 1 |
| Calliphoridae | 2 |
| Cecidomyiidae | 88 |
| Ceratopogonidae | 10 |
| Chironomidae | 15 |
| Chloropidae | 3 |
| Diadocidiidae | 2 |
| Dolichopodidae | 6 |
| Drosophilidae | 1 |
| Empididae | 3 |
| Ephydridae | 2 |
| Fanniidae | 3 |
| Heleomyzidae | 2 |
| Hybotidae | 8 |
| Keroplatidae | 2 |
| Lauxaniidae | 1 |
| Limoniidae | 4 |
| Milichiidae | 1 |
| Muscidae | 16 |
| Mycetophilidae | 38 |
| Pediciidae | 2 |
| Phoridae | 26 |
| Pipunculidae | 3 |
| Psychodidae | 8 |
| Rhagionidae | 2 |
| Rhinophoridae | 1 |
| Sarcophagidae | 7 |
| Scatopsidae | 1 |
| Sciaridae | 32 |
| Sepsidae | 1 |
| Simuliidae | 1 |
| Sphaeroceridae | 4 |
| Syrphidae | 13 |
| Tachinidae | 9 |
| Tipulidae | 2 |
